# Declining Prevalence of Disease Vectors Under Climate Change

**DOI:** 10.1038/srep39150

**Published:** 2016-12-16

**Authors:** Luis E. Escobar, Daniel Romero-Alvarez, Renato Leon, Manuel A. Lepe-Lopez, Meggan E. Craft, Mercy J. Borbor-Cordova, Jens-Christian Svenning

**Affiliations:** 1 Department of Fisheries, Wildlife and Conservation Biology, University of Minnesota, St. Paul, Minnesota, 55108, USA; 2 Department of Veterinary Population Medicine, University of Minnesota, St Paul, Minnesota, 55108, USA; 3 Facultad de Medicina Veterinaria y Zootecnia, Universidad de San Carlos de Guatemala, Guatemala City, Guatemala; 4 Unit of Molecular Parasitology & Tropical Medicine, Centro de Biomedicina, School of Medicine, Universidad Central del Ecuador, Quito, Ecuador; 5 Laboratorio de Entomología Médica & Medicina Tropical, Universidad San Francisco de Quito, Quito, Ecuador; 6 Faculty of Marine Sciences, Biology, Oceanic Sciences and Natural Resources, Escuela Superior Politécnica del Litoral, Guayaquil, Ecuador; 7 Section for Ecoinformatics & Biodiversity, Department of Bioscience, Aarhus University, DK-8000 Aarhus C, Denmark

## Abstract

More than half of the world population is at risk of vector-borne diseases including dengue fever, chikungunya, zika, yellow fever, leishmaniasis, chagas disease, and malaria, with highest incidences in tropical regions. In Ecuador, vector-borne diseases are present from coastal and Amazonian regions to the Andes Mountains; however, a detailed characterization of the distribution of their vectors has never been carried out. We estimate the distribution of 14 vectors of the above vector-borne diseases under present-day and future climates. Our results consistently suggest that climate warming is likely threatening some vector species with extinction, locally or completely. These results suggest that climate change could reduce the burden of specific vector species. Other vector species are likely to shift and constrain their geographic range to the highlands in Ecuador potentially affecting novel areas and populations. These forecasts show the need for development of early prevention strategies for vector species currently absent in areas projected as suitable under future climate conditions. Informed interventions could reduce the risk of human exposure to vector species with distributional shifts, in response to current and future climate changes. Based on the mixed effects of future climate on human exposure to disease vectors, we argue that research on vector-borne diseases should be cross-scale and include climatic, demographic, and landscape factors, as well as forces facilitating disease transmission at fine scales.

More than half of the world’s population is at risk of vector-borne diseases^1^, causing public health concerns due to elevated mortality and high levels of disability-adjusted life-years (DALYs)^2^. Vector-borne diseases are currently a major problem in tropical developing countries^1^. Climate variability influences vector population dynamics, distribution, and vector-borne disease transmission^3^. Dengue transmission is associated in space and time with local climate effects on survival of its vector *Aedes aegypti*—more rain and higher temperature generates more transmission^4^,^5^. Changes in inter-annual climate variability such as the El Niño Southern Oscillation have been shown to be important drivers for malaria transmission^6^. Further, vectors also show distributional shifts from low to high altitudes during warm years^7^.

It has been estimated that by 2100 the average global temperature will have risen between 1.0 and 3.5 °C, radically increasing the burden of vector-borne diseases^8^. As an example, simulations of sandfly vector ecology suggest that leishmaniasis will also shift its potential distribution in response to climate change^9^. Thus, analyzing vector distribution patterns is crucial for providing insights to estimate their present-day distribution in detail and to anticipate their distribution in face of future climate scenarios.

Vectors species’ potential distribution can be explored via ecological niche modeling^10^,^11^. Ecological niche models aim to characterize the environmental conditions required by a species to maintain populations in the long term without need of immigration. Defining these conditions allows us to identify the potential distribution of the species^12^. Models calibrated using available present-day data can also be projected into future climate scenarios^12^.

Ecuador has a considerable burden of vector-borne diseases across a variety of climatic conditions, human population density, and biodiversity across the country (Supplementary Material). Here, we used ecological niche modeling to determine the present-day and future potential distribution of multiple important disease vector species in Ecuador (Table 1). Niche models were estimated using a coarse-grained macroecological approach focused on the broader-scale climate relations of the vectors (e.g., maximum temperature, annual precipitation)^13^. We searched for environmental overlap between niche models of vectors and environments present in Ecuador (Fig. 1 Risk A and B). Niche models were then transferred to future climates to identify future suitable areas for vector presence (Fig. 1 Risk A and C). The geographic areas suitable for the vectors in Ecuador are considered here to be of potential risk for human exposure to the vectors (Fig. 1).

## Results

### Vectors species distribution forecasts

**Figure 2:**
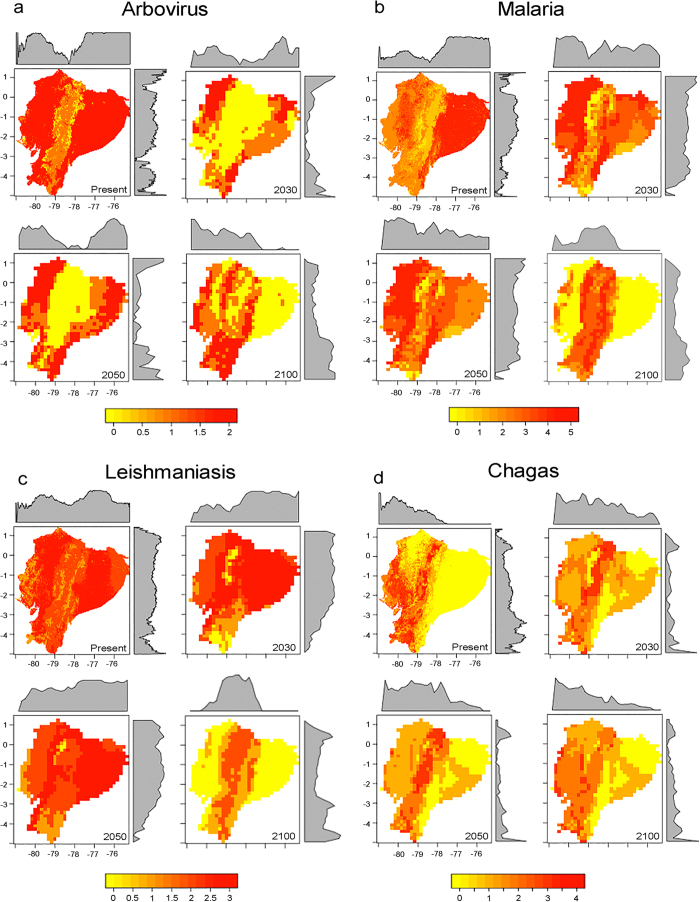
Maps of potential transmission risk in Ecuador. Vector species were grouped by disease. (**a**) arboviruses (chikungunya, dengue, yellow fever, and zika); (**b**) malaria; (**c**) leishmaniasis; and (**d**) chagas disease. Hotspots of vector prediction suggested areas of high (red) or low (yellow) disease transmission risk. (Figure done using the raster and rasterVis packages in R version 3.3.1: A Language and Environment for Statistical Computing, R Core Team, R Foundation for Statistical Computing, Vienna, Austria (2016) https://www.r-project.org).

**Figure 3:**
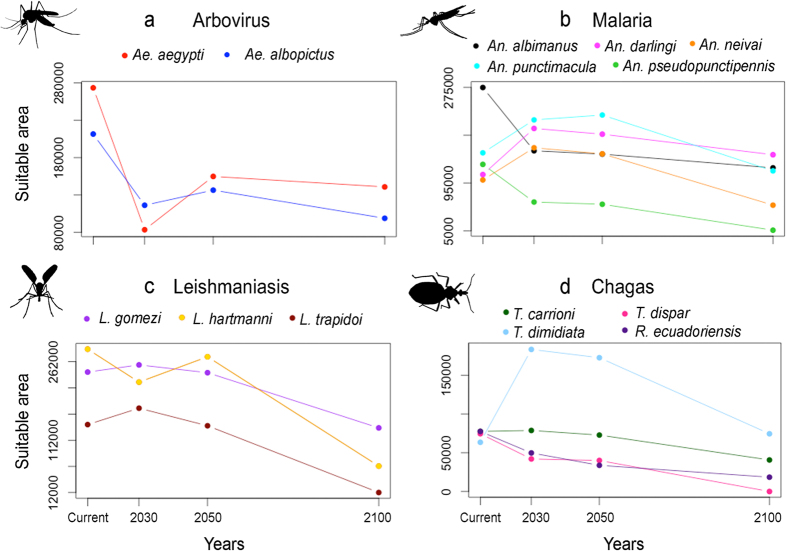
Potential vectors’ distribution under current and future climate scenarios. Number of pixel cells (1 km; *y* axis) predicted suitable by period (*x* axis) for vector species of arboviruses (**a**), malaria (**b**), leishmaniasis (**c**), and chagas disease (**d**) were estimated under current and future climate conditions by 2030, 2050, and 2100.

**Figure 4:**
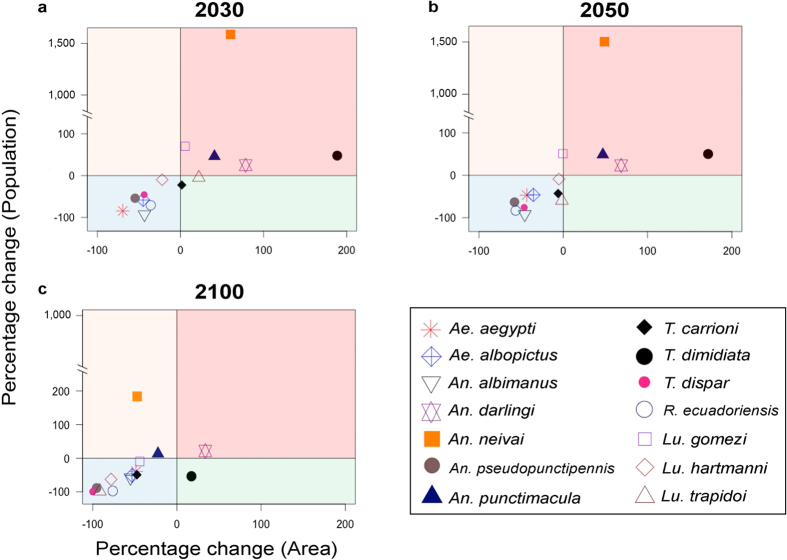
Percent change of vector potential distribution and potential for human exposure to vectors under present-day and future climate. Present-day models were compared against future climate by 2030 (**a**), 2050 (**b**), and 2100 (**c**) in terms of area (*x* axis) and human population at risk of exposure (*y* axis). Top right quadrant (pink) denotes an increase in vectors’ range and human population at risk. Bottom-right (green) resembles an increase in range, but reduction in the number of humans exposed. Bottom-left (blue) denotes reduction in vectors’ range and reduction in population predicted at risk. Top-left (yellow) denotes increase in human populations at risk, but reduction in the vector’s range. Zero means no change. (Plots done using the raster package in R version 3.3.1: A Language and Environment for Statistical Computing, R Core Team, R Foundation for Statistical Computing, Vienna, Austria (2016) https://www.r-project.org).

Our ecological niche models were able to accurately characterize environmental conditions for each vector (Supplementary Fig. S1). The results show that present-day predictions based on remote sensing data reveal areas of high risk for exposure to arboviruses vectors across the coastal region of western Ecuador and in the Amazonian lowlands in the northeast. Additional areas of exposure risk to vectors were found across valleys of the western region of the Andes mountains (Fig. 2), matching dengue endemic areas of Ecuador and reports of the recent chikungunya epidemic^14^. The highlands exhibited reduced suitability to arbovirus vectors under present-day environmental conditions (Fig. 2). Climate-based predictions suggest an increased risk in southern areas in the short term (i.e., 2030). Mid-term predictions suggest that by 2050 high risk will be distributed in the coast, southern lowlands and in northeastern areas neighboring Colombia and Peru, with reduced suitability in central areas across the Andes. Models for 2100 for the arbovirus vectors *Ae. aegypti* and *Ae. albopictus*, showed an evident geographic shift in the suitable areas, with increased risk for exposure in the Andes valleys. *Ae. aegypti* should experience a reduction in its potential area of distribution by 69%, 43%, and 48% and population at risk by 84%, 47%, 40% by 2030, 2050, and 2100, respectively. For *Ae. albopictus*, the potential area of distribution should be reduced by 45%, 35%, and 53% and the number of people potentially exposed by 58%, 46%, and 52% in 2030, 2050, and 2100, respectively (Figs 3 and 4).

Under present-day climate conditions, malaria vectors showed two foci of risk for vectors exposure in west central and northeastern Ecuador. However, under future climates these vectors may find wide suitable areas in western Ecuador across the coast with an impressive shift to the highlands by 2100, resulting in a rise in exposure risk across the Andes Mountains (Fig. 2). The potential area for distribution for *An. albimanus* is forecasted to be reduced by 43%, 46%, and 55% and the estimated people exposed to be reduced by 93%, 92%, and 58% in 2030, 2050, and 2100, respectively (Figs 3 and 4).

Leishmaniasis vectors showed a broad distribution across all the biomes of Ecuador, with higher risk in the lowlands. Models predicted a rise in leishmaniasis exposure risk in the Andes Mountains by 2030 and 2050 (Fig. 2). Surprisingly, by 2100, we found that future climate will provide suitability for leishmaniasis vectors across the Andes, but we were unable to assess future suitability in lowland ecosystems given the non-analogue high temperatures anticipated in such areas (Supplementary Fig. S2).

Vectors of chagas disease commonly occupied highland biomes (e.g., *T. carrioni* and *T. dispar*); thus, under future climate models we forecast extirpation of endemic vector species across Ecuador (e.g., *T. dispar*; Fig. 3 and Supplementary Fig. S1) in face of temperatures rising above those experienced across species current range (Supplementary Fig. S2). *Triatoma dimidiata* is forecasted to increase its potential distribution by 189% in 2030, 172% in 2050, and ~17% in 2100 (Fig. 3).

### Vector exposure under climate change

The general pattern revealed that human populations in the Andean highlands would be increasingly exposed to disease vectors as the future climate changes unfold due to likely upward vector species range shifts (Fig. 2 and Supplementary Fig. S3). While models in the Northern Hemisphere show a displacement of species from south to north and models in the Southern Hemisphere show displacements from north to south^9^,^15^, we found that the equatorial latitudes of Ecuador show just a slight northward range shift, with strong upward altitudinal displacements into the Andes Mountains. Importantly, across all 14 vector species we found consistent non-artefactual reductions in potential range area and people potentially exposed for nine vector species by 2030, 2050, or 2100, with the malaria vectors *Anopheles darlingi* and *An. neivai* as the main exceptions (Figs 3 and 4; and Supplementary Fig. S1). The synchrony between reduction of areas and populations at risk among the numerous vectors explored, revealed a pattern of potential adverse ecological effects of climate change on vector distributions (Fig. 3). Indeed, three species showed reduction of both area and people potentially exposed under all three future climate time steps (*Ae. aegypti, Ae. albopictus, An. albimanus*; Figs 3 and 4; and Supplementary Fig. S1). Lack of future suitable environments was found for two vector species, suggesting potential for extinction (i.e., *T. dispar*, and *An. pseudopunctipennis*, Supplementary Fig. S2).

## Discussion

Controlling calibration area, novel climates, model complexity, and model transference to ensure robust predictions, we developed forecasts of geographic shifts in the potential range under future climates for 14 important disease vectors in Ecuador, finding consistent predictions of reductions in the vector’s potential ranges and the number of people exposed for most of the species studied. Our modeling experiments were developed based on a detailed design supported by ecological theories while outputs were evaluated carefully^12^. The overall patterns suggest that vectors of arboviruses and leishmaniasis will experience geographic range reductions by 2100 under future climate conditions, while chagas vectors had mixed results with some species increasing (*T. dimidata*) and others reducing (*T. carrioni, T. dispar, R. ecuadoriensis*) their geographic distribution (Fig. 3). A similar situation was observed for malaria vectors where one species (i.e., *An. darlingi*) was predicted to increase in the geographic range while the other species were forecasted to experience range reductions. Therefore, *An. darlingi* and *T. dimidata* are of particular public health concern as such vectors could expand their range under future climate conditions, which could impact the epidemiology of malaria and chagas disease in Ecuador respectively. Climate change was even forecasted to possibly extirpate important vectors such as *Triatoma dispar*, a vector of chagas disease. A reliable extinction would require ecological niche conservatism with no adaptation of vectors to warming climates; adaptation of species to warming climate is, however, restrained by high temperatures^16^,^17^. In other words, there is a physiological upper limit that cannot be exceeded easily by species, providing some confidence to our estimation. Our results show a benefit of climate change in terms of plausible extinction and range reduction for some vectors and consequent decrease in exposure risk for people, contrasting with the proposed negative effects of climate change on human health^18^. Our findings support other mathematical modeling and laboratory experiments proposing potential benefits of climate change in terms of limiting the burden of malaria and dengue vectors^19^,^20^,^21^,^22^, mitigating potential overinterpretation of other studies suffering incorrect study designs and thus proposing dramatic impacts of future climate on the epidemiology of infectious diseases which could fail to inform decision makers with realistic scenarios. Studies showing the attenuated negative impacts on the epidemiology of some vector species may help to reduce focus on the effects of climate change on the burden of vector-borne diseases of likely reduced future importance, and may encourage scientists to instead focus on the effects of other equally or more important factors for future human health, such as land use change, to help in the design and implementation of effective public health policies. We argue that a mature understanding of climate change effects on health could be achieved if and only if the research communities moves to a broader interpretation of climate effects, covering plausible negative and positive effects of such changes across ecosystems and taxa.

We have assumed static patterns of human density in Ecuador in face of climate change, in other words, given that we based our estimation on values of human density, we assume that the present-day patterns of low and high will remain in the future. However, human displacement as a result of climate change is a complex factor that deserves further exploration. Further, topographic steepness limits the potential for upslope human migrations in the region, with most non-steep upland areas already densely occupied. While there is consensus that vectors are sensitive to weather and climate^23^, there is still uncertainty on the various impacts of climate variability on fine-scale transmission dynamics and socioeconomic variables^23^,^24^. We mitigated uncertainty in our forecasts by tuning model parameters for biologically realistic predictions by capturing the environmental tolerances of vectors from their entire geographic range and assessing model fit with available data. Our present-day predictions highlight areas of vector suitability at high spatial resolution for all the species included in this study. These areas suitable for vectors occurrence should be considered into account by public health authorities given the plausible disease underreporting in Ecuador (i.e., epidemiological silence^14^). The areas predicted to be of present-day risk may be of special interest for testing control measures and for identifying currently neglected human populations. The areas found suitable via remote sensing data could be also useful to guide local studies aiming to understand the distributional ecology of vectors, considering their interaction with other vector species, prey, variations in microclimate, availability of breeding sites, and the effects of socio-economic factors facilitating vectors’ occurrence^25^. Such local-scale factors were not considered in this study, but would provide valuable information to better understand fine-scale patterns in the ecology of vectors across different geographic and temporal scales^26^. An inter-sectoral approach should be implemented at the municipality level to address issues of sanitation, safe water availability, and preventive health care in areas predicted of present-day risk (Fig. 2).

Our models were based on a broad-scale biogeographic approach^27^, therefore we did not consider microclimatic conditions at local scale and avoided the complexities and uncertainties of including factors such as biological interactions in the modeling^12^. We accounted for the environmental tolerances of vector species from vector records across the entire global distribution of each species. This framework should reduce the risk of incomplete niche estimation (niche truncation) due to failure to represent the full niche space for a species in the model calibration^28^, i.e., by including records from as wide areas in geographic and environmental space as possible (e.g., the entire species range). Thus, for species of broad distributions (e.g., global), making models with data from the areas of interest only (e.g., Ecuador) could capture a limited portion of the species’ ecological niche generating an incomplete characterization of the species’ environmental tolerances. Models calibrated using a subset of the species’ range will generate sub-estimations of the species’ ecological niche and in turn sub-estimations of its potential distribution.

The future climate forecasts used in this study (A2; 2030, 2050, 2100) are realistic scenarios of the environmental conditions that may be present in Ecuador given the ongoing trends in greenhouse gas emissions^29^. Predictions in future climate scenarios showed a likely expansion of exposure risk of all pathogens to populations in the highlands of Ecuadorian Andean regions. The geomorphology and latitudinal position of Ecuador may suggest similar predictions for other tropical regions. Although potential range shifts and extinction have been reported for the future distribution of several plant and animal species^30^, we complement this information with the potentially negative effects of climate change on range contraction, displacement, and extirpation of vectors species, and an overview of changes in patterns of human populations at risk for Ecuador. These estimates were based on the assumption of niche conservatism of vector species^31^. By supporting this assumption, our findings provide information to anticipate strategies to reduce the burden of vector-borne diseases via increasing the awareness in areas currently disease-free, but predicted vulnerable to vectors displacement. An early warning scheme in Andean region should be formulated with inclusion of non-endemic vector species given risk predicted in highlands and the limited understanding of the colonization capacity of vectors in face of climate change. Preventive measures can contribute globally to the early detection and prevention of vector-borne diseases in highlands worldwide in face of global warming. Despite the local benefits of reduced transmission due to the extinction of specific vector species, range shifts of other vectors could increase vector-borne diseases incidence and spread, resulting in epidemiological surprises^32^. Based on our mixed results of positive and negative effects of future climate on human exposure to disease vectors, we argue that research on vector-borne diseases in face of future environmental change should be cross-scale and include climatic, demographic, and landscape factors, as well as forces facilitating disease transmission acting at fine scales. Importantly, care should be take that vector-borne disease research on climate change effects will not discourage health agencies from research into understanding local transmission processes and implementing control measures for critical threats of disease transmission at the local level including human behavior and habitat destruction.

## Methods

We forecasted the distribution of disease vectors under present-day conditions and future climate in Ecuador using vector records across their entire global ranges from data of museum collections and literature. After vector occurrence data curation, we developed ecological niche models for each vector species in Maxent^33^,^34^, assuming that each species record originated from a stable vector population instead of migratory or accidentally translocated individuals. We assessed regularization coefficients and model complexity in Maxent^34^,^35^. Our model evaluation was based on information theory through assessing Akaike information criterion (AIC) values to explore model fit with the data available^35^, which provides robustness to our model assessment procedure, a key factor when modeling infectious diseases^10^. Thus, AIC is a well-known method to discriminate among models, especially when working with the Maxent algorithm employed in our study^35^. Additionally, using Maxent we estimated the Area Under the Curve of the Receiver Operating Characteristic values as this metric is commonly employed to assess the specificity and sensitivity of models. The final models, calibrated on each species’ entire range, were projected to Ecuador under present-day and future climatic conditions using strict model transference^36^,^37^. Potential distribution of vectors were forecasted under present-day conditions using remote sensing imagery including EVI time series data and land surface temperature^38^, and precipitation values from field stations^39^, while future climate models where calibrated in present-day climate and then projected to future climate conditions, based on data of the CliMond repository^39^. We used the SRES A2 scenario (Special Report on Emissions Scenarios) of future climatic conditions given that Latin America has increasing populations, economic development, per capita economic growth, but also relatively fragmented and slow technological change and limited abilities to mitigate greenhouse effects^40^, and considering that the last IPPC assessment concluded that there are not important improvements to reduce emissions worldwide^41^. Indeed, since the first future climate scenarios, the more optimistic low-emission scenarios have become implausible given the ongoing empirical trends in emissions^29^,^42^,^43^. The A2 scenario is equivalent to the Representative Concentration Pathway (RCP) 8.5 proposed in the IPCC Fifth Assessment Report (AR5)^41^. Additionally, we developed non-analogous environment evaluations using the Mobility Oriented Parity test (MOP) script in R^37^ to ensure that future predictions were restricted to regions in Ecuador with analogous climates somewhere in range of each species, to avoid extrapolation uncertainties. The final maps were used to identify the percentage of area predicted as suitable in each climate scenario. We used the LandScan human population estimates for year 2011 at 1-km spatial resolution^44^ to estimate the people living within the suitable areas for a given vector species. Estimation based on current climates was compared with models of future climate conditions to establish the percent of change among climate scenarios. A detailed description of the modeling framework is available in the Supplementary Material.

## Additional Information

**How to cite this article**: Escobar, L. E. *et al*. Declining Prevalence of Disease Vectors Under Climate Change. *Sci. Rep.* **6**, 39150; doi: 10.1038/srep39150 (2016).

**Publisher's note:** Springer Nature remains neutral with regard to jurisdictional claims in published maps and institutional affiliations.

## Supplementary Material

Supplementary Material

Supplementary Data

## Figures and Tables

**Figure 1:**
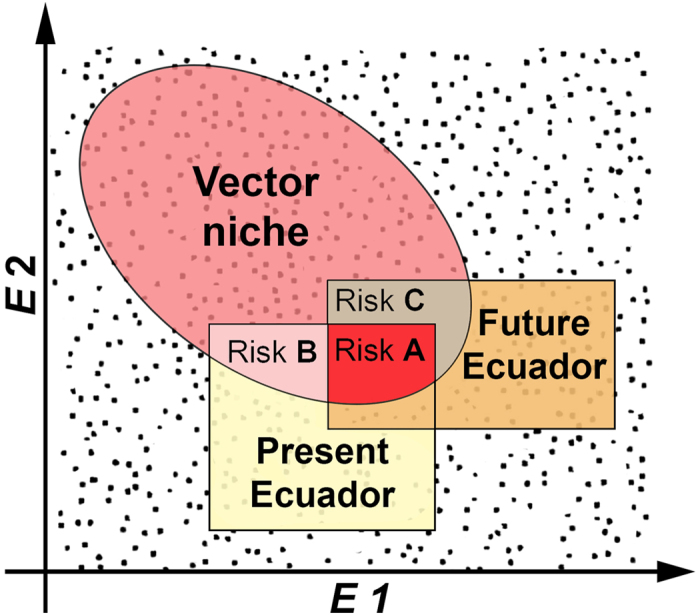
Ecological modeling approach used to assess disease vector species’ potential distributions. The ecological niche of each vector species was estimated (red ellipsoid) based on present-day environmental conditions (*E*). The environments available in Ecuador were determined under present-day (yellow square) and future (orange square) climate conditions. Areas with climatic conditions in Ecuador overlapping between the niche of vectors and both present-day and future climate conditions were identified as Risk A (risk under present-day and future scenario; red). Areas of Ecuador with climate under present-day overlapping with the niche of vectors were defined as Risk B (risk under present-day only; pink). Areas with overlap between the niche of vectors and future climate in Ecuador only were defined as Risk C (risk under future climate only; brown). (Figure created using Adobe Photoshop CC 2014 https://www.adobe.com).

**Table 1:** List of vector species by disease included in this study.

Disease	Pathogen		Vector		
	Agent	Genus	Common name	Order	Vector species
Chagas disease	Protozoa	*Trypanosoma*	Kissing bug	Hemiptera	*Rhodnius ecuadoriensis*
					*Triatoma carrioni*
					*T. dimidiata*
					*T. dispar*
Chikungunya	Virus	*Alphavirus*	Yellow fever mosquito	Diptera	*Aedes aegypti*
			Asian tiger mosquito		Ae. albopictus*
Dengue Fever	Virus	*Flavivirus*	Yellow fever mosquito	Diptera	*Aedes aegypti*
			Asian tiger mosquito		*Ae. albopictus*
Malaria	Protozoa	*Plasmodium*	Anopheles mosquito	Diptera	*Anopheles albimanus*
			American malaria mosquito		*An. darlingi*
			Mosquito		*An. neivai*
			Mosquito		*An. pseudopunctipennis*
			Mosquito		*An. punctimacula*
Leishmaniasis	Protozoa	*Leishmania*	Phlebotomine sandfly	Diptera	*Lutzomyia gomezi*
					*Lu. hartmanni*
					*Lu. trapidoi*
Yellow Fever	Virus	*Flavivirus*	Yellow fever mosquito	Diptera	*Ae. aegypti*
			Asian tiger mosquito		*Ae. albopictus*
Zika	Virus	*Flavivirus*	Yellow fever mosquito	Diptera	*Ae. aegypti*

^*^Not reported officially in Ecuador and not active surveillance for its monitoring, but present in neighboring countries.
