# Supplementary material for: Declining Prevalence of Disease Vectors Under Climate Change
Source: Sci Rep. 2016 Dec 16;6:39150. doi: 10.1038/srep39150 (PMC5159793; doi:10.1038/srep39150)
Supplement: Supplementary Material [file srep39150-s1.pdf]

Declining Prevalence of Disease Vectors Under Climate Change

Luis E. Escobar*, Daniel Romero-Alvarez, Renato Leon, Manuel A. Lepe-Lopez,

Meggan E. Craft, Mercy J. Borbor-Cordova, Jens-Christian Svenning

*To whom correspondence should be addressed: E-mail: lescobar@umn.edu

How to cite this article: Escobar, L. E. *et al.* Declining Prevalence of Disease Vectors Under Climate Change. *Sci. Rep.* **6**, 39150; doi: 10.1038/srep39150 (2016).

This Supplementary Information contains:

- Supplementary Fig. S1: Binary models of vectors by disease
- Supplementary Fig. S2 : Comparison of climates between present-day climate in study areas **M** and future conditions in continental Ecuador
- Supplementary Fig. S3 : Consensus maps of overall human risk of exposure to disease vectors in Ecuador
- Supplementary Material
- References

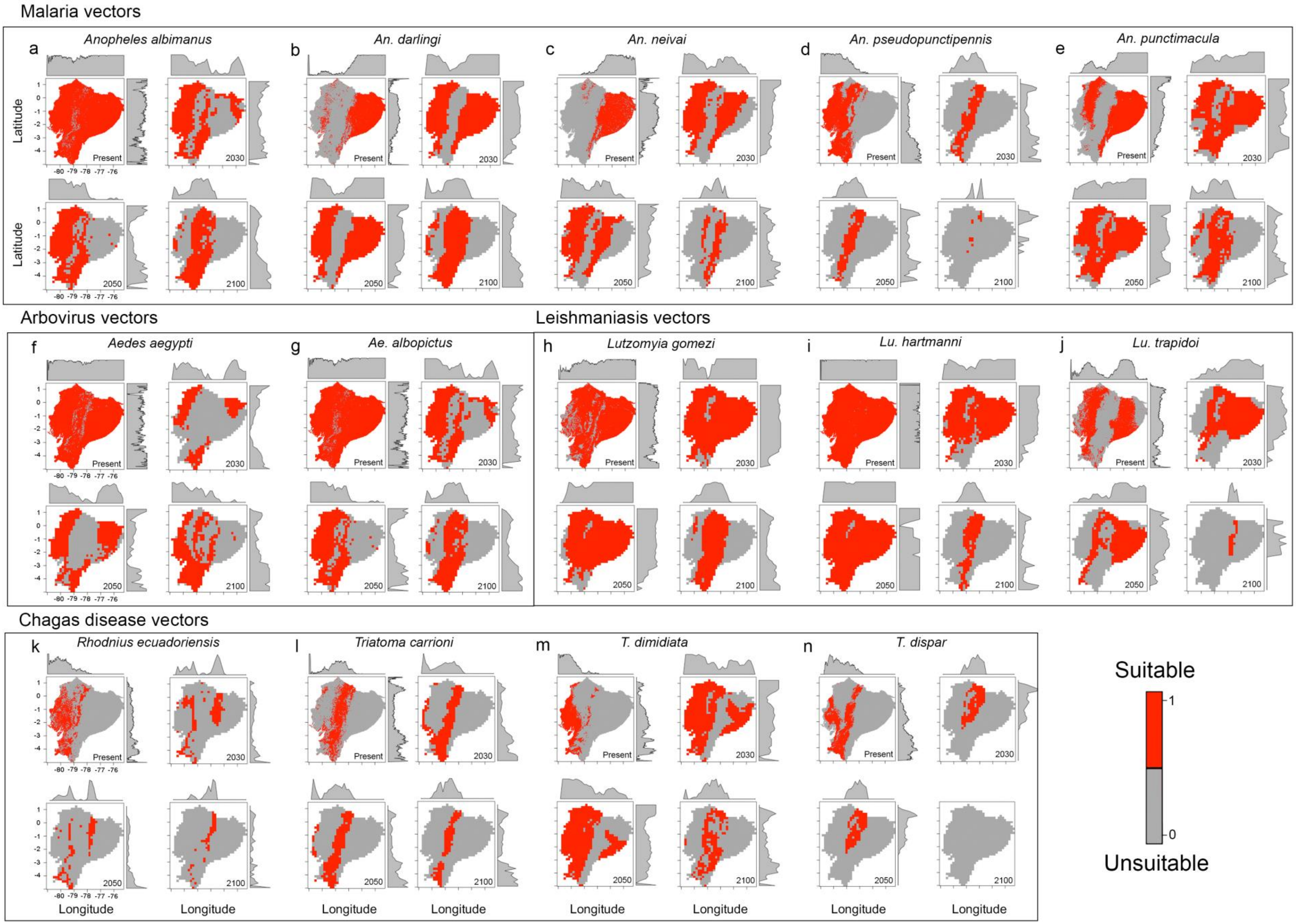

Malaria vectors
a
Anopheles albimanus
b
An. darlingi
c
An. neivai
d
An. pseudopunctipennis
e
An. punctimacula
Latitude
Present
2030
2050
2100
Arbovirus vectors
f
Aedes aegypti
g
Ae. albopictus
Leishmaniasis vectors
h
Lutzomyia gomezi
i
Lu. hartmanni
j
Lu. trapidoi
Chagas disease vectors
k
Rhodnius ecuadoriensis
l
Triatoma carrioni
m
T. dimidiata
n
T. dispar
Longitude
Suitable
Unsuitable

Figure S1. Binary models of vectors by disease. Model calibrated in the study area "**M**" was transferred to continental Ecuador under present-day and future climate conditions by 2030, 2050, and 2100 for vectors of malaria (*An. albimanus* (**a**), *An. darlingi* (**b**), *An. neivai* (**c**), *An. pseudopunctipennis* (**d**), *An. puntimacula* (**e**)), arbovirus (*Ae. aegypti* (**f**) and *Ae. albopictus* (**g**)), leishmaniasis (*Lu. gomezi* (**h**), *Lu. hartmanni* (**i**), *Lu. trapidoi* (**j**)), and chagas (*Rhodnius ecuadoriensis* (**k**), *T. carrioni* (**l**), *T. dimidiata* (**m**), *T. dispar* (**n**)). (Maps done using the raster and rasterVis packages in R version 3.3.1: A Language and Environment for Statistical Computing, R Core Team, R Foundation for Statistical Computing, Vienna, Austria (2016) https://www.r-project.org)

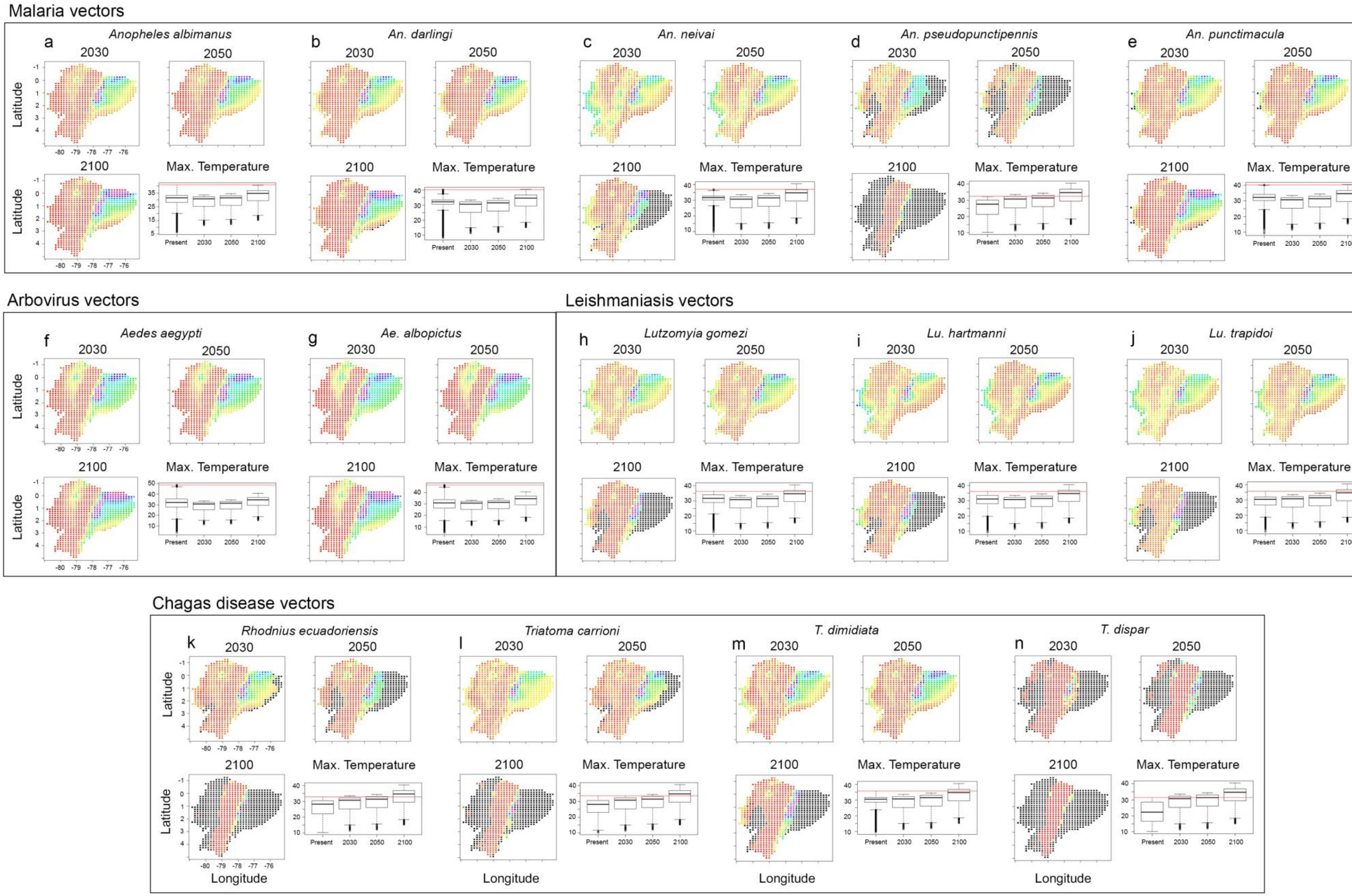
Malaria vectors
a
Anopheles albimanus
b
An. darlingi
c
An. neivai
d
An. pseudopunctipennis
e
An. punctimacula
2030
2050
2100
Max. Temperature
Latitude
Present
Arbovirus vectors
f
Aedes aegypti
g
Ae. albopictus
Leishmaniasis vectors
h
Lutzomyia gomezi
i
Lu. hartmanni
j
Lu. trapidoi
Chagas disease vectors
k
Rhodnius ecuadoriensis
l
Triatoma carrioni
m
T. dimidiata
n
T. dispar
Longitude

Figure S2. Comparison of climates between present-day climate in study areas M and future conditions in continental Ecuador. *An. albimanus* (**a**), *An. darlingi* (**b**), *An. neivai* (**c**), *An. pseudopunctipennis* (**d**), *An. puntimacula* (**e**), *Ae. aegypti* (**f**), *Ae. albopictus* (**g**), *Lu. gomezi* (**h**), *Lu. hartmanni* (**i**), *Lu. trapidoi* (**j**), *Rhodnius ecuadoriensis* (**k**), *T. carrioni* (**l**), *T. dimidiata* (**m**), *T. dispar* (**n**). Mobility-Oriented Parity (MOP) analysis showing non-analogous climates (black cells) and Multivariate Environmental Similarity Surface (MESS) test showing high (red) and low climatic similarity (blue) for 2030, 2050, and 2100. Box plots of maximum temperature of the hottest week identify temperature of Ecuador inside (below the red line) or outside (above the red line) the range of temperature across the vectors' range (**M**) in present-day and future climate periods. (Maps done using the raster package in R version 3.3.1: A Language and Environment for Statistical Computing, R Core Team, R Foundation for Statistical Computing, Vienna, Austria (2016) https://www.r-project.org)

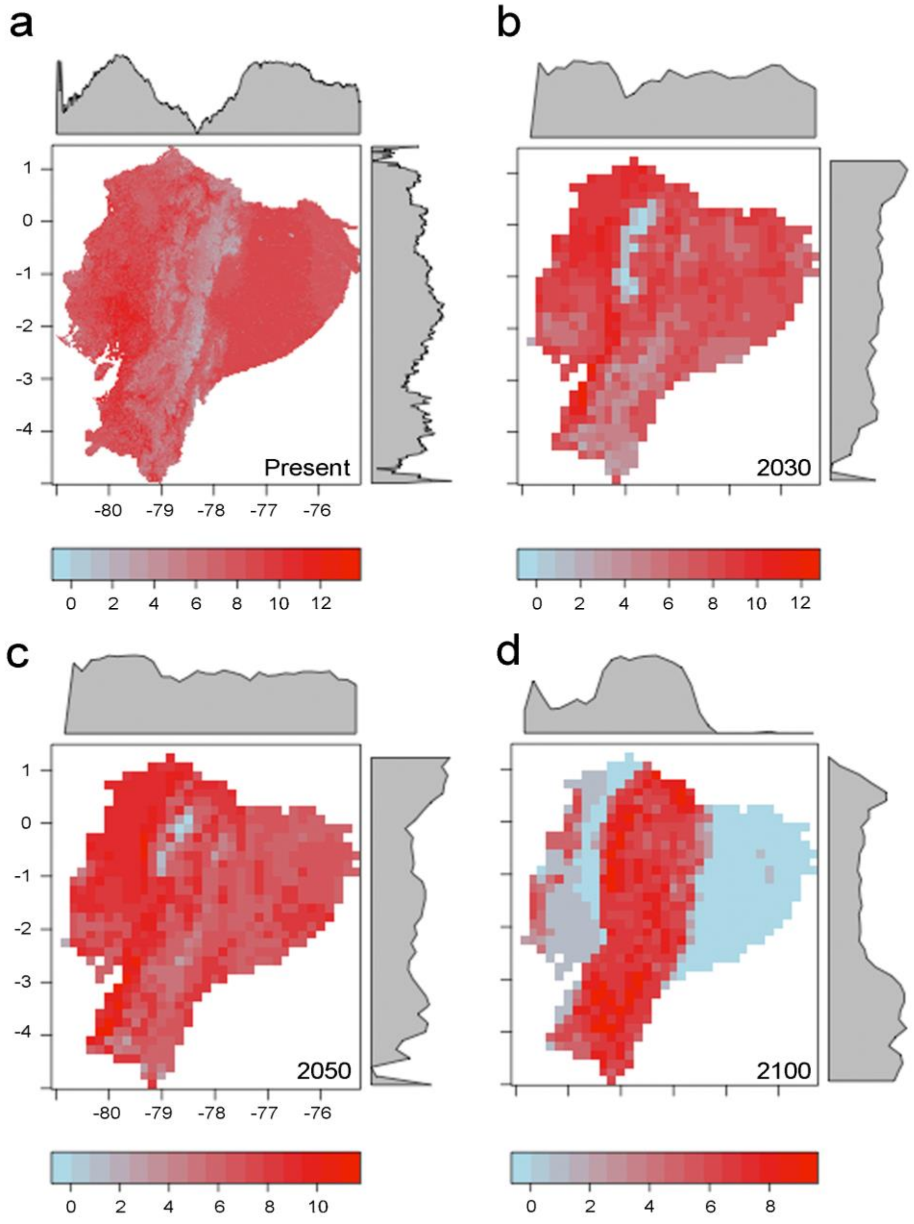

Figure S3. Consensus maps of overall risk of exposure to disease vectors in Ecuador. Assemble of binary vector models, under present-day (**a**) and future climate conditions by 2030 (**b**), 2050 (**c**), and 2100 (**d**), to identify areas of high (red) or low (blue) potential vector occurrence. (Maps done using the raster and rasterVis packages in R version 3.3.1: A Language and Environment for Statistical Computing, R Core

Team, R Foundation for Statistical Computing, Vienna, Austria (2016) https://www.r-project.org)

Supplementary Material: Methods

Dengue

Dengue fever is the most widely distributed vector-borne disease in Latin America and the Caribbean; from 2000 to 2010 dengue was reported by 40 countries and territories in the Americas[1,2]. Dengue virus is a mosquito-borne positive stranded RNA arbovirus, grouped into five serotypes[3]. Globally, *Aedes aegypti*, commonly known as the yellow fever mosquito, is the primary vector species; however, other *Aedes*, such as *Ae. albopictus,* the Asian tiger mosquito, are also important vectors of dengue[4]. Dengue fever in Ecuador was mostly controlled by the 1950's through intensive vector control strategies, nevertheless, the disease re-emerged in 1988 and is currently endemic in the country with a variable spatial and seasonal distribution[5,6]. Dengue epidemiology shows annual peaks of hemorrhagic cases, from hundreds to thousands of cases per year (Fig. S4). In Ecuador, data of dengue without signs of alarm or non-hemorrhagic dengue ("dengue sin signos de alarma") revealed more than 15,000 cases in 2010 and 13,865 cases in 2014[5]. In the period between January and October 2015, 42,483 cases of dengue were reported in Ecuador[7], and by September 2016, at least 12,571 cases have been reported[8]. Most of these dengue infections occurred in the subtropical region of the country, including the Amazon region, the Pacific coast, and the Galapagos islands; a low number of cases were imported from tourism and traveling populations[7,5].

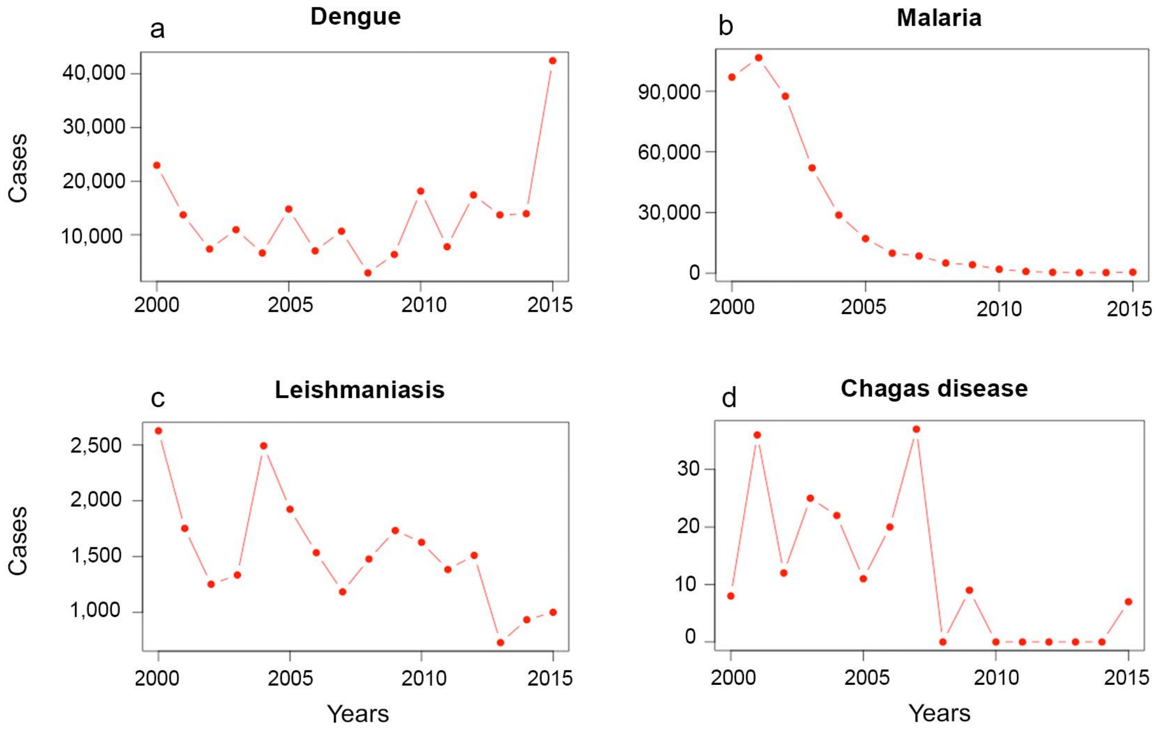

Figure S4. Case reports of vector-borne diseases in Ecuador by October 2015. Note the consistent incidence of dengue fever and recent epidemic (**a**), the sustained reduction on malaria reports (**b**), an increasing in leishmaniasis cases is observable in 2015 (**c**), and reduction of chagas disease incidence since 2010 (**d**). Source: [7,5]. (Plots done using R version 3.3.1: A Language and Environment for Statistical Computing, R Core Team, R Foundation for Statistical Computing, Vienna, Austria (2016) https://www.R-project.org)

Leishmaniasis

Leishmaniasis is a neglected disease in Ecuador reported since the beginning of the 20th century and has been recorded from 21 of the 24 Ecuadorian provinces[9]. To date, at least eight *Leishmania* spp. have been identified to infect humans and non-human mammals[10,11]. Leishmaniasis reports in rural areas occur from sea level to ~2,700 m

elevation[11]. More than 60% of all *Leishmania* species of Ecuador are reported in the subtropical and tropical lowlands of the Pacific region (Fig. S5), where *Le*. *panamensis* and *Le. guyanensis* are the most common. In the highlands of the Andes region in central Ecuador (Fig. S5), the main leishmaniasis agents are *Le. mexicana* (>80%) and *Le. major*-like[11–14]. In this country, leishmaniasis reached 2,628 cases by 2000, decreasing to 935 cases by 2014[5]. By May 2016, ~1,400 cases of leishmaniasis were reported[15] (Fig. S4).

Leishmaniasis is transmitted by infected phlebotomine sandflies of the genus *Lutzomyia*. From the approximately 81 sandfly species reported in Ecuador, only a few, including *Lu. trapidoi* and *Lu. gomezi* have been incriminated as disease vectors[16,17]. In the Andes foci of *Leishmaniasis*, six anthropophilic *Lutzomyia* (*hartmanni, gomezi, nevesi, ayacuchensis, serrana,* and *osornoi*) have been identified to date[9].

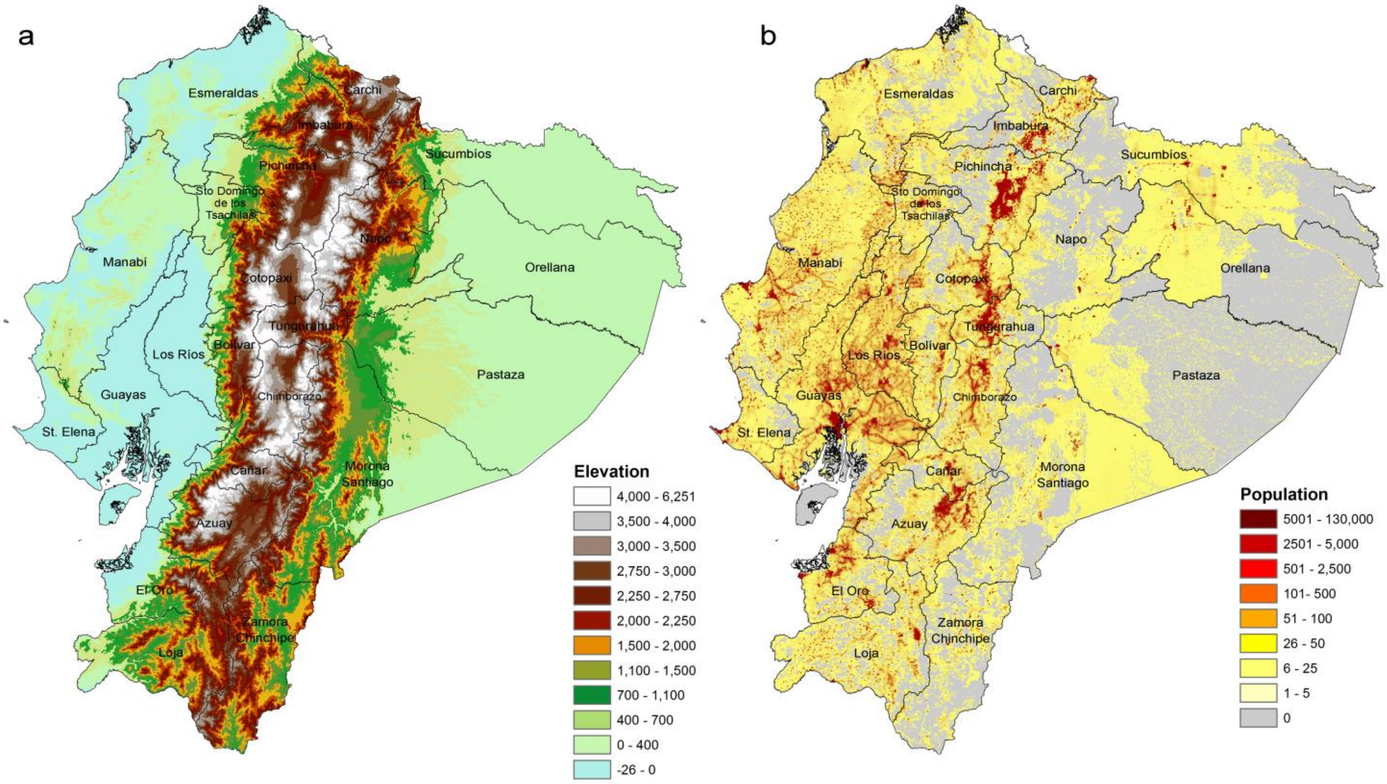

Figure S5. Topographic and population features of continental Ecuador. a) Elevation map. Notice the highlands in central Ecuador (white) representing the Andes Mountains. **b**) Population distribution at one-kilometer resolution cells across Ecuador by 2011 LandScan data [Ref. [18]]. (The population map was made utilizing the LandScan 2011™ High Resolution global Population Data Set copyrighted by UT-Battelle, LLC, operator of Oak Ridge National Laboratory under Contract No. DE-AC05-00OR22725 U.S. Department of Energy. The United States Government has certain rights in this Data Set. Neither Ut-Battelle, LLC nor the U.S. Department of Energy, nor any of their employees, makes any warranty, express or implied, or assumes any legal liability or responsibility for the accuracy, completeness, or usefulness of the data set). (Maps created using ArcGis, Envrionmental Systems Research Institute, version 10.2, Redlands, CA (2016) http://www.esri.com/)

Chikungunya

Chikungunya is an arboviral disease with symptoms somewhat similar to dengue fever, characterized by sudden onset of fever, rash, and severe joint pain lasting until 14 days; some cases result in persistent arthritis even 25 days after initial symptoms[19]. At the end of 2014, the first imported case of chikungunya virus was reported in Ecuador and less than a year later, the number of locally transmitted cases raised to ~30,000 since the start of the epidemic to October 2015[7]. Strikingly, the number of chikungunya cases is likely underreported in the country[20] with 7.5 % of cases established by epidemiological link. Additionally, the eminent risk of the introduction of other arboviruses to Ecuador is an emergent public health concern; for example, by September 2016 zika virus has been detected in at least 2,000 human patients in several coastal provinces of the country[21]. Yellow fever is nowadays a disease of less concern, but still present in Ecuador[22].

Malaria

Malaria is still the most important vector-borne disease worldwide transmitted by *Anopheles* mosquitoes[23] with reports of up to 80,000 cases/year in Ecuador. Interestingly, the country has been reporting most cases of dengue and few cases of malaria in the last five years[24] (Fig. S4), a pattern that changes the current public health priority from malaria to dengue[25]. This trend has been poorly addressed and might be associated with efficient vector control programs, reduction in epidemiological surveillance, or other factors including climate variability reducing vector abundance as

fewer cases of malaria have been reported even in regions lacking Anopheline mosquitoes control. However, malaria is still of importance in Ecuador given the potential for re-emergence, the persistence of Anopheline mosquitoes in rural areas where the disease was endemic, the mortality associated with infection, and DALYs[23]. By May 2016, 229 cases of malaria were reported in Ecuador[15].

Chagas

Chagas disease is another vector-borne disease historically endemic in the Andean region and in Ecuador where it is still a cause of morbidity and mortality (Fig. S4 and S5). It is transmitted by the feces of infected triatomine bugs and can cause chronic infections with permanent damage to several organs including the heart, the esophagus, and the colon. From the approximately 16 species of triatomine bugs reported in Ecuador only *Triatoma dimidiata* and *Rhodnius ecuadoriensis* and a few others have been incriminated as vector of chagas disease[26]. Vector eradication campaigns driven by the central government have largely controlled vector populations in cities as Guayaquil and Machala in southern Ecuador in the past, but transmission continues in residual endemic foci[27–29], while the transmission at the Amazon basin has been poorly investigated[30–32]. By October 2015, seven cases of chagas disease were reported in Ecuador[15].

The framework of our modeling approach included a detailed characterization of the study area, occurrences, and model fit for each vector species under present-day

environmental conditions derived from satellite imagery and future climate. A summary of the methodology is found in Figure S6 and is explained in detail below.

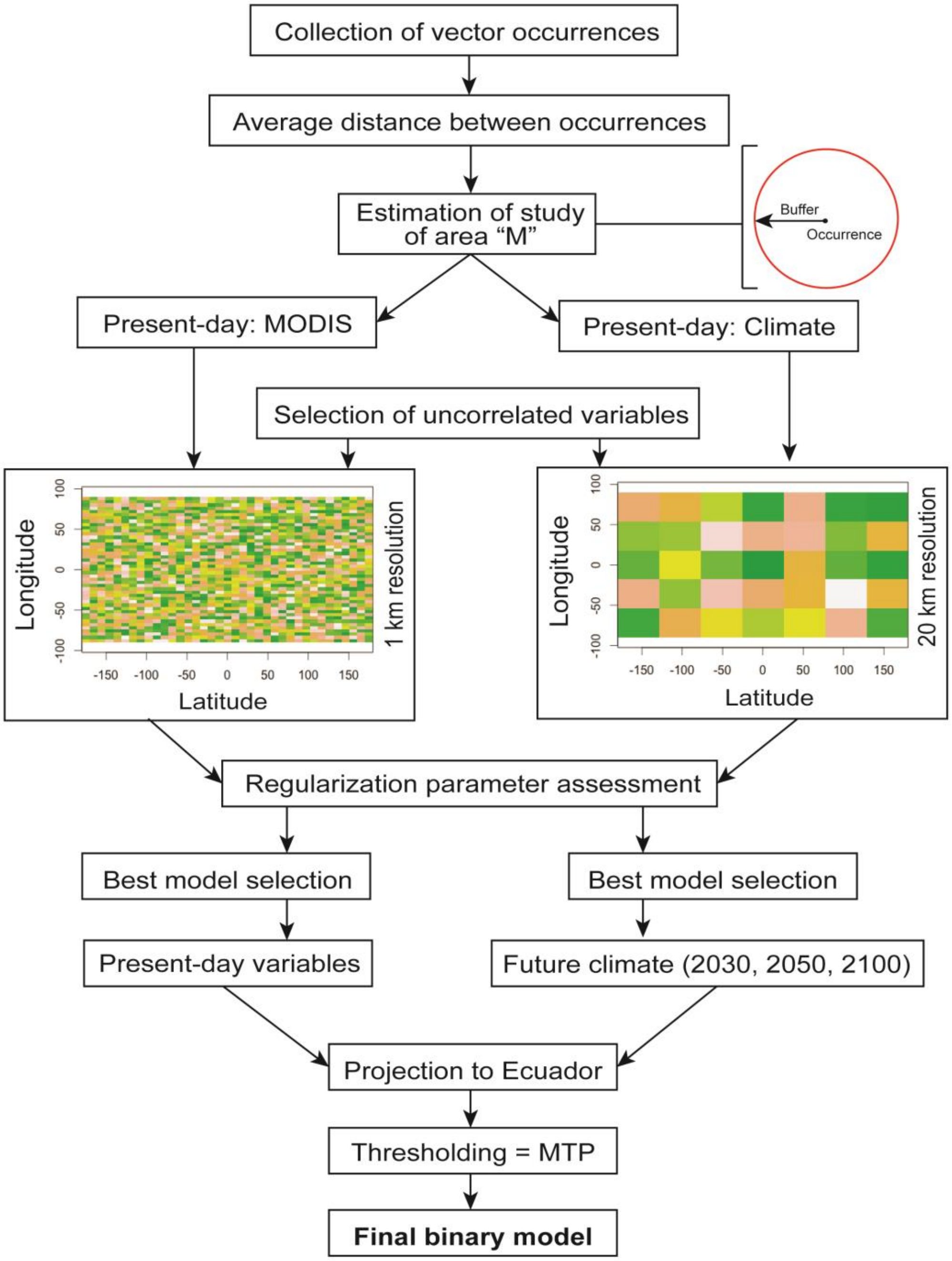

Figure S6. Modeling frameworks employed in this study. Models of vector species were developed using satellite imagery and climate data. Final predictions were

converted to binary maps. Binary maps were then used to identify hotspots areas of potential risk of exposure to vectors. MTP: Minimun training presence. (Grids done using the raster package in R version 3.3.1: A Language and Environment for Statistical Computing, R Core Team, R Foundation for Statistical Computing, Vienna, Austria (2016) https://www.R-project.org <https://www.r-project.org>)

Vector occurrence

We determined the environmental conditions necessary for vectors using geographic coordinates of vector reports and environmental variables under present-day environmental conditions, then, the existence of such environmental conditions was determined across Ecuador (Fig. 1 of the manuscript). We selected vector species associated with six vector-borne diseases recognized in Ecuador including malaria, chikungunya, dengue, leishmaniasis, chagas disease, and yellow fever, as well as zika fever. The vector species corresponding to each disease included mosquitoes, kissing bugs, and sandflies (Table 1 of the manuscript).

To obtain vector occurrence data, we conducted a review from March to April 2015 in open access biodiversity datasets at the Global Biodiversity Information Facility[33] and SpeciesLink[34] networks with data provided by natural history museums collections from field captures and identification of species by experts[35]. Museum collection providing such data included: Centro de Pesquisa René Rachou (Coleção de Mosquitos Neotropicais CMN-Fiocruz); University of Puerto Rico (Invertebrate Collection); Field Museum of Natural History (Zoology), Insect, Arachnid, and Myriapod Collection;

European Molecular Biology Laboratory (EMBL); Lyman Entomological Museum (LEMQ) from McGill University; Walter Reed Biosystematics Unit, Smithsonian Institution; Ibáñez Bernal, S. 1998 de Yucatán, México; and María Cristina Mayorga Martínez museum, Instituto de Biología, UNAM. A vector occurrence was represented as geographic coordinates of a single vector report. Complementary occurrences for unrepresented species (i.e., <5 occurrences) were obtained from a review of scientific articles searched in Google Scholar, SCIELO, and PubMed. Keywords were the scientific name of the vector species e.g. "*Rhodnius ecuadoriensis*;" we reviewed literature in both Spanish and English to identify site locations of the species report[36–42]. We removed the duplicate occurrences by species to obtain single occurrence points by site and to reduce model overfitting due to oversampled areas[43] (Supplementary Dataset). The vector occurrence records were then used to develop ecological niche estimations, under the assumption that each record originated from a stable population that can persist without need of immigration.

Areas for model calibration

A critical step in the niche modeling process is the selection of areas for model calibration[43]; the extent of such areas dramatically impacts the area predicted as suitable for vector occurrence[44]. The study design of ecological niche models should be based on biogeographic features for each vector species[44]. For this, study designs should follow the **BAM** framework (*sensu* Soberón and Peterson[45]). This framework is used in modern ecology during the study design and interpretation of ecological niche modeling. The **BAM** framework identifies three factors that interact in the species'

ecological niche delimitation: **B**: biotic; **A**: abiotic; and **M**: dispersal capacity. Under this framework and in view of the considerable effects of the study area extent in model results[44], modelers should establish specific geographic areas for model calibration for each species according to the species' dispersal capacity **M**.

Considering the impact of the study area extent on ecological niche model predictions and the need for biological realism in the study design[43,44], models should be calibrated in areas representing a proxy of **M**[44]. We assumed that each species' **M** could be estimated based on the average geographic distance among vector occurrences[46]. Briefly, for each vector species we estimated a centroid point from all the occurrences of the vectors' distribution and measured the distance between the centroid to all the occurrences (Table S1). We focused on the vectors' native range to avoid over estimation in transoceanic dispersal of the invasive vectors of the *Aedes* genus. The average geographic distance between the centroid and occurrences was used to generate a buffer zone where models were calibrated (Fig. S7). This method provides impartiality from the modeler to establishing the extent of the calibration area, furthermore it is based on the potential dispersal of the species providing biological meaning to model results, and allows the characterization of the environments occupied by species across their distribution[44]. For the estimation of the average distance between occurrences of *Ae. aegypti* and *Ae. albopictus*, we used occurrences in the native ranges of Africa and South Asia respectively[47]. For all other species, we estimated the average distance restricted to reports of their native distribution in the Americas.

Supplementary Table S1. Distance among vector occurrences in the geographic space (geographic degrees).

Vector species	Mean
Aedes aegypti (Linnaeus 1762)	23.27
Anopheles albimanus (Wiedemann 1821)	10.34
Aedes albopictus (Skuse 1895)	13.50
Anopheles darlingi (Root 1926)	13.19
Anopheles neivai (Howard Dyar & Knab 1914)	5.41
Anopheles pseudopunctipennis (Theobald 1901)	1.31
Anopheles punctimacula (Dyar & Knab 1906)	5.33
Lutzomyia gomezi (Nitzulescu 1931)	3.84
Lutzomyia hartmanni (Fairchild & Hertig 1957)	3.21
Lutzomyia trapidoi (Fairchild & Hertig 1952)	1.32
Rhodnius ecuadoriensis (Lent & Leon 1958)	1.32
Triatoma carrioni (Larrousse 1926)	1.37
Triatoma dimidiata (Latreille 1811)	7.37
Triatoma dispar (Lent 1950)	0.67

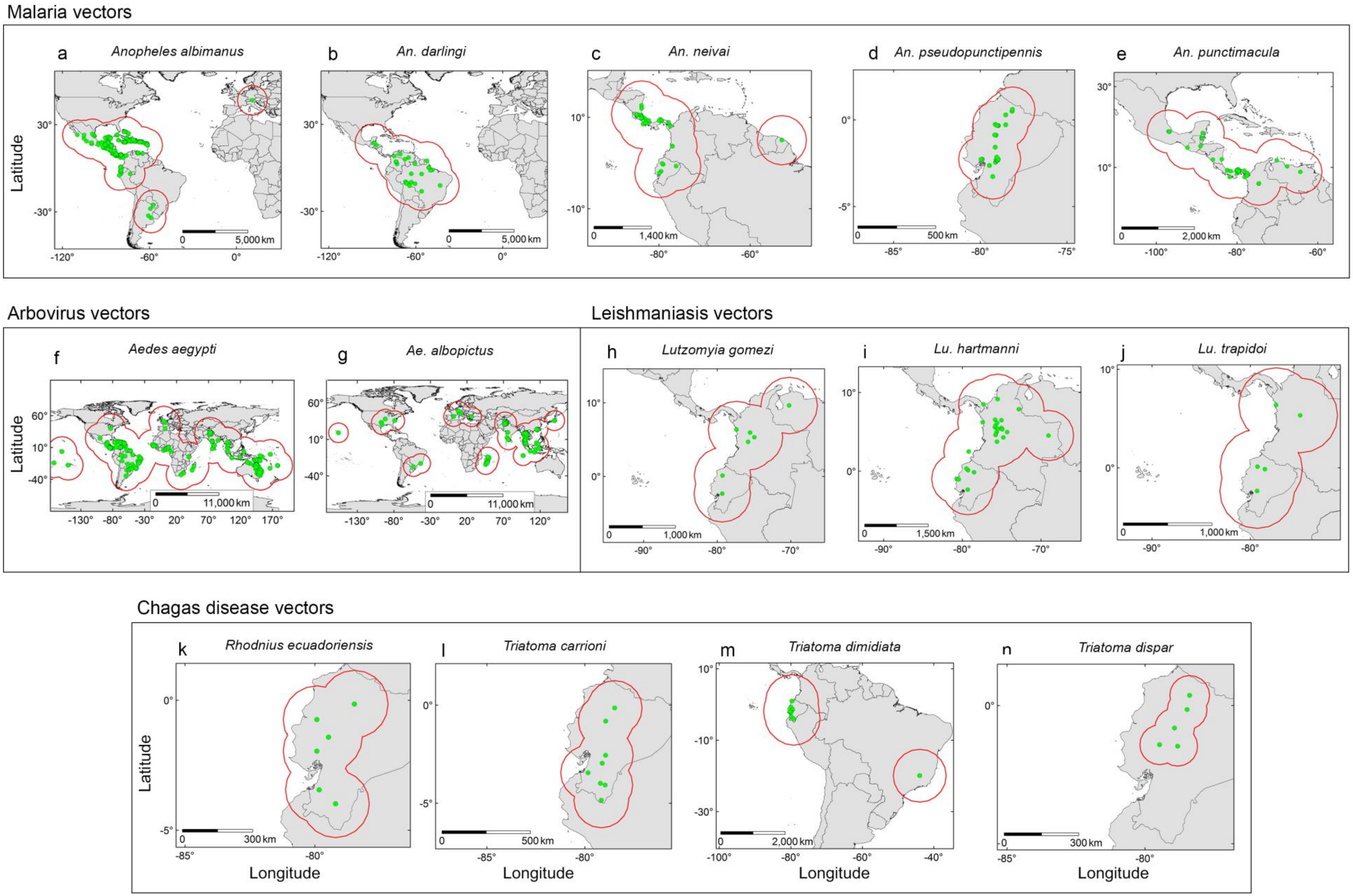
Malaria vectors
a Anopheles albimanus
b An. darlingi
c An. neivai
d An. pseudopunctipennis
e An. punctimacula
Latitude
5,000 km
1,400 km
500 km
2,000 km
Arbovirus vectors
f Aedes aegypti
g Ae. albopictus
11,000 km
Leishmaniasis vectors
h Lutzomyia gomezi
i Lu. hartmanni
j Lu. trapidoi
1,000 km
1,500 km
Chagas disease vectors
k Rhodnius ecuadoriensis
l Triatoma carrioni
m Triatoma dimidiata
n Triatoma dispar
300 km
2,000 km
Longitude

Figure S7. Study area "M "used in this study for model calibration based on the species' potential dispersal. Average distances between points for *Ae. aegypti* and *Ae. albopictus* were estimated based on occurrences from their native area of distribution. Average distances were employed to generate a buffer zone (red line) around occurrences (green points). (Figures created using ArcGis, Envrionmental Systems Research Institute, version 10.2, Redlands, CA (2016) http://www.esri.com/)

Present-day environmental variables

We generated ecological niche models using environmental variables with biophysical relevance in vectors biology[48]. For our present-day estimations, we used environmental variables in the form of satellite imagery from the Moderate-resolution Imaging Spectroradiometer (MODIS) at ~1 km spatial resolution from the WorldGrids repository[49]. Original variables included the maximum, mean, minimum, and standard deviation values of land surface temperature values of 8-days composites; one data set captured at daytime and another at nighttime during 2011-2012, mean and standard deviation values of the monthly MODIS enhanced vegetation index (EVI) during time series data for 2001-2012, and long-term MODIS-estimated Evapotranspiration during 2000-2012. Additionally, we included long-term precipitation for four periods comprising i) November, December, and January; ii) February, March, and April; iii) May, June, and July; and iv) August, September, and October with time series data (1950-2000) from the Worldclim repository[50]. We used the software NicheA version 3.0 to identify correlation between environmental variables[51], and removed layers with high correlation (>0.8) and important gaps of data in Ecuador (i.e., >1% area of the country).

Climate change environmental variables

Models for future climate were calibrated based on historical climate data and then projected to a future climate scenario. We used climate data from version 1.2 of bioclim variables from the Climond repository at 10' spatial resolution[52]. Present-day climate variables are composed by historical data from 1961-1990. For the future climate scenarios, we used the A2 model, as proposed by the Intergovernmental Panel on Climate Change (IPPC), including scenarios for years 2030, 2050, and 2100[52]. This future climate scenario was selected considering the consistent use of fossil fuel with no global agreement to reduce greenhouse gas emissions[52]. Previous conservative models of future climate, especially the B family (i.e., B1, B2), have been proposed as implausible considering recent emissions records[53–55]. The A2 model from the SRES scenario is equivalent to the 8.5 model from the RCP scenario. Indeed, RCP 8.5 was based on the socio-economic and demographic background, assumptions, and technological approach of the IPCC A2 SRES[56]. In practical terms, A2 values fall between RCP 6 and 8.5 [Ref. [57,58]]. Climate variables included bioclimatic variables from Bio 1 to Bio 35[52,59]. We removed highly correlated variables (>0.8) and selected those that, according to us, have high biological relevance for vector species[60]. For realistic interpretations of models under future climate conditions[61,62], we assessed environmental similarity between vectors present-day and future climate distribution in Ecuador via Mobility-Oriented Parity (MOP) and Multivariate Environmental Similarity Surface (MESS)[61,63] analyses in R[64].

Ecological niche modeling

Ecological niche models were developed using Maxent software version 3.3.3.k[65]. Maxent uses a logistic regression-like algorithm which associates environmental variables with vector occurrences (for a detailed explanation of algorithms used by Maxent see[66,67]). Several models were developed for each species to obtain the best model fit as follows. First, models for each species were calibrated under present-day climate conditions using different regularization coefficients. Low regularization coefficients generate model overfit to the conditions of the location of vector occurrence, while a high regularization coefficient allows overprediction. To identify the model with the best fit with the data, we explored models using ten parameter levels in Maxent including 0.5, 1, 1.5, 2, 2.5, 3, 3.5, 4, 4.5, and 5 [Ref. [68]]. Model fit was measured according to Akaike information criterion (AIC) values using the ENMTools software version 1.4.4 [Ref. [69]]. Given the high number of occurrences, *Ae. aegypti* and *Ae. albopictus* models using remote sensing data were developed using a regularization coefficient of 1.

Models with the highest performance, based on regularization coefficient evaluations were used to build final models for each species in the calibration areas **M** and were later transferred to continental Ecuador under present day and future environmental conditions. Areas with environmental overlap between the vector's niche and environments available in Ecuador were defined as areas of "risk of exposure to disease vectors," based on the environmental suitability for vector species (Fig. 1 in the manuscript). Maxent settings for the final models included median of the logistic output,

random seed, 100 bootstrap permutations, and clamping and extrapolation turned off for an strict model transference in Maxent[63,69], in view of the perilous predictions in novel environments when extrapolation and clamping are allowed in the algorithm[61,62,70]. In sum, we generated 220 models by each species for a total of 3,080 models for all species, including present and future climate conditions. From the 100 model permutations, we estimated the mean values of the area under the curve (AUC) of the of the receiver operating characteristic to evaluated models in terms of sensitivity and specificity[65]. Final models were converted to binary using a threshold based on minimum training presence (Fig. S6). These maps were used to estimate suitable areas by vector on present-day climates and people living in these suitable areas. We estimated overall patterns of people density exposed to vectors under current climate using 1 km resolution of human population in Ecuador from LandScan[18]. We employed this data set also to assess the population at risk under future conditions considering the agreement of LandScan with future human population models from Ecuador[71] ($r^2 = 0.43$, $p < 2 \times 10^{-16}$). We then estimated the area predicted suitable for the vectors. Finally, we estimated the percent of change of area suitable for the vector species and population exposed to vectors between present-day and future climate scenarios.

Supplementary Material: Results and Discussion

Occurrences

In all, 1,184 single occurrences were obtained from the 14 vector species included in this study (Supplementary Data). We obtained 421 occurrences for *An. albimanus,* 288 for *Ae. aegypti,* 267 for *Ae. albopictus,* 35 fo *An. neivai,* 49 *An. punctimacula,* 29 for *An. darlingi,* 28 for *Lu. hartmanni,* 26 for *An. pseudopunctipennis,* 10 for *T. dimidiata,* 8 for *T. carrioni,* 7 for *Lu. gomezi,* 6 *R. ecuadoriensis,* 5 for *Lu. trapidoi,* and 5 for *T. dispar*. Most species were reported from more than one country, except for *An. pseudopunctipennis, R. ecuadoriensis, T. carrioni, and T. dispar* reported in Ecuador only (Fig. S7). The continent with most occurrences for a single species' was the Americas for *An. albimanus* and *Ae. aegypti*. The Americas covered the entire range for 11 of the 14 vector species modeled.

Present-day models

The selected uncorrelated variables for our present-day models were mean and standard deviation of the monthly EVI time series data; long-term precipitation for two periods including i) November, December, and January; and ii) May, June, and July; maximum value and standard deviation of the 8-day MODIS day-time LST time series data; and the mean value and standard deviation of the 8-day MODIS night-time LST time series data. We did not include minimum and mean value of the 8-day MODIS day-time LST time series data, long-term MODIS-estimated evapotranspiration, and the mean value of the long-term lights at night images, as they have important gaps in data

in continental Ecuador. For the future climate models environmental variables were annual mean temperature (°C), mean diurnal temperature range (mean[period max-min],°C)), annual precipitation (mm), precipitation seasonality (%), precipitation of warmest quarter (mm), annual mean radiation (W m^{-2}), radiation seasonality (%), radiation of wettest quarter (W m^{-2}), and radiation of warmest quarter (W m^{-2}).

The **M** estimation for *Ae. aegypti* represented the broader study area, with occurrences in five continents (Fig. S7). *Ae. albopictus* followed in geographic extent with an **M** distributed across Africa, the Americas, Asia, and Europe; in Africa the species' occurrence was restricted to Madagascar island. *An. albimanus* occurrences extended its **M** to the Americas and Europe. *An. neivai* extended its **M** from northwest South America (i.e., Colombia, Ecuador, and northern Peru) to Honduras. *An. punctimacula* extended from northern Colombia and Venezuela to southern Mexico. **M** estimations for *Lu. gomezi* and *Lu. hartmanni* included Colombia, Ecuador, Panama and Venezuela, whereas *Lu. trapidoi* included southern Panama, northern Colombia, Peru and Ecuador. In the case of *An. pseudopunctipennis*, *R. ecuadoriensis*, and *T. carrioni*, **M**s included the Andean region of Ecuador, southern Peru, and northern Colombia. *T. dispar* was restricted to Ecuador (Fig. S7).

According to our AIC evaluations, the best regularization coefficients based on remote sensing data and precipitation ranged between 0.5 and 3, the most frequent values were 1 and 2, while for models based climate for future conditions, parameters ranged between 0.5 and 5, the most frequent was 0.5 (Table S2). There was a negative

association between the number of parameters and AIC values (r^2 range = 0.42 - 0.93; $p<0.05$), with the exception of *R. ecuadoriensis* when calibrated using climate. We did not find associations between the number of occurrences and the regularization coefficient value of the best Maxent models calibrated using remote sensing (r^2 = 0.31; p=0.07) nor climate (r^2 = 0.18; p=0.15). We found associations between the lowest AIC values and lowest regularization coefficients in 67% of the vector species modeled using remote sensing and in 64% of the species modeled using climate (Table S2). The AUC evaluations showed a good discriminatory capacity of models to predict occurrences better than a random model (Table S3).

Supplementary Table S2. Akaike information criterion (AIC) values for model selection. AIC values for models based on present-day MODIS + precipitation variables at ~1 km and present bioclimatic variables at ~19 km. The best models (bold) were selected according to lowest AIC values. Number of parameters are shown in parenthesis. x = evaluation excluded (e.g., more parameters than occurrences).

Species	Regularization coefficient									
**MODIS + precipitation ~1 km**	**0.5**	**1**	**1.5**	**2**	**2.5**	**3**	**3.5**	**4**	**4.5**	**5**
Anopheles albimanus	**1,0937.45**	10,956.94	10,991.50	11,048.04	11,065.12	11,083.63	11,068.54	11,096.27	11,103.9	11,099.96
An. darlingi	x	x	x	880.85	**877.32**	878.06	880.53	883.63	889.53	896.02
An. neivai	x	x	1,017.41	1,006.96	1,004.47	**1,003.37**	1,029.9	1,008.84	1,005.21	1,009.53
An. pseudopunctipennis	x	610.44	609.65	**604.39**	606.46	610.39	614.74	619.55	622.9	631.06
An. punctimacula	x	1,324.91	1,306.4	**1,291.37**	1,295.1	1,294.51	1,298.42	1,301.98	1,299.63	1,303.79
Lutzomyia gomezi	203.97	204.57	203.86	**202.28**	200.31	200.31	200.31	200.31	200.31	200.31
Lu. hartmanni	x	723.57	719.67	725.5	724.96	**719.45**	723.78	726.36	726.11	729.22
Lu. trapidoi	141.43	**140.89**	139.93	139.93	139.93	139.93	139.93	139.93	139.93	139.93
Rhodnius ecuadoriensis	x	**145.44**	150.39	149.49	148.08	148.27	148.46	148.67	148.89	149.11
Triatoma carrioni	**191.63**	195.06	197.71	199.53	202.59	201.41	201.8	202.23	200.61	200.63
T. dimidiata	x	**286.32**	291.82	291.33	291.82	292.43	294.08	296.11	296	296.2
T. dispar	x	x	112.65	112.31	113.23	**111.76**	112.03	112.31	112.6	112.89
**CliMond ~19 km**										
Ae. aegypti	**5,588.95**	5,645.31	5,635.58	5,633.59	5,650.18	5,642.46	5,653.82	5,660.49	5,648.95	5,656.28
Ae. albopictus	**4,404.84**	4,511.08	4,577.26	4,614.39	4,661.02	4,667.47	4,667.56	4,668.11	4,673.65	4,676.71
Anopheles albimanus	**6,507.90**	6,593.58	6,640.90	6,664.24	6,689.72	6,707.87	6,744.19	6,762.24	6,789.82	6,797.16
An. darlingi	x	x	x	626.58	623.72	626.63	613.40	**596.10**	600.44	600.87
An. neivai	x	x	**887.39**	889.62	893.52	903.82	919.45	927.76	930.63	943.20
An. pseudopunctipennis	x	x	326.47	320.64	**316.19**	317.60	318.06	321.91	327.51	329.25
An. punctimacula	x	x	x	x	801.16	778.53	775.23	779.66	766.41	**761.67**
Lutzomyia gomezi	117.99	117.10	118.25	**116.97**	117.47	117.95	117.99	117.95	117.24	117.51
Lu. hartmanni	x	x	464.54	460.96	457.81	466.38	468.73	456.85	454.62	**452.17**
Lu. trapidoi	x	83.27	82.53	**81.12**	81.62	82.08	x	x	x	x
Rhodnius ecuadoriensis	**81.76**	x	x	x	x	x	x	x	x	x
Triatoma carrioni	108.36	108.44	107.64	106.62	107.52	106.29	107.02	**105.39**	105.71	106.02
T. dimidiata	**166.58**	174.92	180.68	178.52	179.46	180.22	178.19	179.42	180.80	180.39
T. dispar	x	x	**57.68**	x	x	x	x	x	x	x

Supplementary Table S3. Area under the curve (AUC) values for model evaluation. Average AUC values for models based on present-day MODIS + precipitation variables at ~1 km and present-day bioclimatic variables at ~19 km.

Final mean AUC		
**Species**	**Remote sensing data**	**Climate data**
Ae. aegypti	0.94	0.97
Ae. albopictus	0.96	0.98
A. albimanus	0.95	0.98
A. darlingi	0.91	0.80
A. neivai	0.85	0.95
A. psuedopunctipennis	0.85	0.82
A. punctimacula	0.88	0.83
L. gomezi	0.75	0.76
L. hartmanni	0.86	0.77
L. trapidoi	0.87	0.83
R. ecuadoriensis	0.89	0.77
T. carrioni	0.92	0.73
T. dimidiata	0.93	0.93
T. dispar	0.81	0.80

When the final models were developed and transferred to Ecuador, broad suitable areas were found across the country for *Ae. albopictus* and *Ae. aegypti*; highlands in central Ecuador limit the potential distribution of these vectors, but *Ae. aegypti* appear to be a more generalist species, tolerating environments available in highest zones (Fig. S1). In contrast to countries located in temperate regions, tropical countries possess only two climatic seasons (i.e., rainy and dry) with high minimum temperatures and rainfall favoring vector species such as *Ae. aegypti* mosquito abundances and lead to the increase of dengue cases during the rainy season[72,73]. A recent global prediction anticipates that the global burden of chikungunya and dengue may increase under future climate scenarios if *Aedes* mosquitoes are considered[74]. Herein, we focused in *Ae. aegypti* and *Ae. albopictus*, as the first is currently reported in Ecuador, and the second is highly abundant in Ecuador's neighboring countries Colombia and Peru. *Ae. aegypti* provided clues about the plausible transmission risk of yellow fever in urban areas and chikungunya and dengue viruses. Additionally, our models may provide information on the critical areas that deserves surveillance in view of the potential spread of zika virus in Ecuador.

Based on vectors suitability, potential areas for malaria occurrence were found in southwestern Ecuador in Los Rios and Guayas, contrasting with the extensive continuous suitable area east to the Andes Mountains involving the major percentage of the three Amazonian provinces Sucumbios, Orellana and Pastaza -from north to south- and areas of Napo and Morona Santiago. The southern and last of the Amazonian provinces of Ecuador, Zamora Chinchipe, has some patches of suitable areas related

with lowlands (Fig. 2 in the manuscript and Fig. S5). Reduced risk of exposure to vectors of malaria in the lowlands in eastern regions of the country, *An. neivai* and *An. pseudopunctipennis*, may be a consequence of novel environments where no predictions were allowed and thus the risk of exposure in such areas was not accounted for these species. Novel environments were particularly evident for *An. pseudopunctipennis*, thus, high uncertainty exist in prediction outside the Andes region; novel environments for this species may reflect temperatures warmer than those in areas where this vector occurs. In Latin America, a displacement from low to highlands was predicted recently for malaria vectors in Colombia[75]. Our results suggest that warming temperatures would displace vector species to the Andes. A previous study using ecological niche modeling also suggested that under climate change, malaria vectors may shift their distributional areas abandoning areas with high human density to invade areas with low human density reducing the overall number of people exposed and in consequence the burden of the disease in Africa[76]. These model results should be considered with caution given that niche models are commonly based on coarse scale patterns, neglecting fine scale population ecology and human interventions to reduce disease prevalence[48].

Models of chagas disease vectors showed a particular and complex pattern of suitability across central-south areas of the country with special emphasis on the coast and Andes (Fig. 2 in the manuscript). All provinces of the coast region were compromised with the exception of the northern province of Esmeraldas, affected to a lesser extent. All the provinces of the Andean region were also compromised and presented suitable

available areas for the vectors, especially in the Andean plateau. On the other hand, eastern Ecuador, at Orellana and Pastaza provinces, presented no suitability for chagas vectors with suitable areas found in other Amazonian provinces (i.e., Napo, Sucumbios, Morona Santiago, and Zamora Chinchipe). Configurations on the potential distribution of chagas disease vectors varied dramatically according to the species involved ranging from highlands in Central Ecuador (*T. carrioni*) to additional adaptations to coastal environments (*T. dimidata*; Fig. S1).

Leishmaniasis vectors were predicted across Ecuador, with special foci of risk in the western slope of the Andes Mountains. The eastern slope of these Mountains also revealed vector suitability, with increased risk in vectors distribution in the Amazonian provinces of Sucumbios, Napo, Orellana, Pastaza, Morona Santiago, and Zamora Chinchipe. Risk of exposure to vectors of leishmaniasis was high in lowlands (Fig. 2 in the manuscript), this pattern was influenced by *Lu. trapidoi*, whereas *Lu. hartmanni* was the leishmaniasis potential vector with the broadest distribution of all species included in the study, finding suitable environments across the country (Fig. S1).

The general pattern of vector potential distribution in Ecuador under present-day conditions, highlights the southwestern area of Los Rios and Guayas as a main foci of risk (Fig. S3). Under present-day environmental conditions, the consensus map of vectors potential distribution showed low vectors richness in the Andes Mountains with high risk mainly present in the valleys between them, in the provinces of Carchi, Imbabura, Pichincha, Cotopaxi, Chimborazo, Tungurahua, Bolivar, and Azuay, from

north to south. Loja province belongs to the Andean region but does not have important elevations (Fig. S5); this area has the highest risk of potential vector distribution in the south (Fig. S3). Vectors distributions were anticipated in areas beyond the records available to us in Ecuador. This pattern would be a result of successful eradication program in some areas where vectors were extirpated, areas with unavailable prey of microhabitats, or could reflect situations in which vectors have suitable conditions in these areas but are unable for successful dispersal and colonization.

Future climate models

Under near future climate (i.e., 2030), patterns of vector occurrence revealed a slight displacement of high risk from southern areas of the country to northwestern Ecuador. In this period, areas of risk increase also in northern Andes (Fig. S3). By 2050, models forecast high risk across the Pacific coast from central to northern areas of Ecuador. Models for 2100 anticipated an abrupt range shift in patterns of risk of exposure to disease vectors, with suitable areas focused in central Ecuador, in the highlands, for most of the vector species (Fig. S3). This future climate scenario showed areas of high risk in northern Ecuador specifically in the provinces Carchi, Imbabura, Santo Domingo de los Tsachilas and small areas of Sucumbios and Pichincha, hosting Ecuador's capital and in turn high human densities. In the middle of the Andean region, Bolivar, Chimborazo, and Morona Santiago represented high risk of potential vector's distribution due to warming climates. In southern Ecuador, high risk areas were predicted under future climate conditions in the provinces of Azuay, Loja, and Zamora Chinchipe. Under the 2100 scenario, some models failed to find suitable conditions in

Ecuador for some vectors (Fig. S2), for example, *T. dispar* may be extirpated due to unsuitable conditions in the future considering the absence of tolerable climate for the species (Fig. S1). However, the comparison between present-day and future climate in Ecuador via Mobility-Oriented Parity test revealed areas with future climate not available in the present-day distribution of this species (Fig. S2). The presence of non-analogous environments, where no prediction was allowed, was particularly evident for species with narrow distributions with maximum temperatures above those available currently in the species range (Fig. S1 and S2).

Our future climate models offer coarse spatial resolution variables (i.e., ~20km) compared with the fine resolution from remote sensing data we employed (i.e., ~1km; Fig. S3). Future climate scenarios have implicit the uncertainty[52], and basing niche models on climate solely does not include the complex biotic interactions among communities that may result in unexpected ecological surprises not captured or forecasted by our models[77]. We are sympathetic with these limitations of climate based models, but also recognize that future climate scenarios provide opportunities to anticipate and adapt to the effect of climate change on human health[78]. Additionally, we employed data of human populations across Ecuador under current climate conditions and explored the risk of this population under future climate. We avoided the use of future human population scenarios considering that the main gain of future population models would be a temporal match between future climate and future population, but at the cost of amplifying uncertainty. In other words, future climate models have considerable uncertainty, thus, using future population models will result in risk

estimations including the uncertainties of both future climate models and future population models, thereby amplifying the uncertainties in the system. We found that patterns of current and future population in Ecuador will remain stable. However, future research exploring the accuracy of essambling future climate with future population models is warranted.

Occurrence data may also contribute to uncertainty from sampling bias[79]. Bias could be generated from oversampling in areas of easy access or from countries with active epidemiological surveillance systems, thus, biased data will result in biased models[79]. To mitigate the effect of bias in model calibration, we utilized binary models instead of continuous models resembling sampling bias effort[43]. Additionally, we employed all the occurrences available to us for each vector species, this allowed us to capture a representative sample of the environmental signature required by each vector species across its known geographic distribution. Models for some species were calibrated from a low number of occurrences, likely reflecting the low reporting effort and data digitalization of Ecuador or the low abundances of such species. Previous niche models of vector species have considered vector reports in the study area only, neglecting vector data from areas outside the areas of interest (e.g., [80–82]). This model design results in models explaining just a limited portion of the species ecological niche, thus, generating pseudomodels that not capture the environmental range tolerated by the vectors[43]. Excluding the entire dataset of existing reports of a vector's distribution, when modeling the ecological niche, i) limits the biogeographic understanding of the species[83,84], ii) has statistical weaknesses[85], and iii) lack of biological interpretation[43,48].

Our study design considering the entire geographic range of vectors increased the information provided to the model to characterize the environmental space occupied by the species and in consequence its environmental tolerances[43], mitigating the plausible environmental bias of the data[79,86].

Our selection of vector species was derived by the known importance of species or their potential role in the disease transmission. However, we may be neglecting other vector species with a role in transmission. For example, from the 81 species of phlebotomine sandflies reported from Ecuador, just a few have been studied in depth. The sandfly species *Lutzomyia trapido*i and *Lu. gomezi* have been incriminated as vectors at the Pacific coast of Ecuador[16,17,87] whereas *Lu. ayacuchensis* have been reported to be a vector at a localized focus at the highlands in Paute canton, a village located at southern section of the Andes Mountains in Ecuador[88]. Similarly, to our knowledge *Lu. hartmanni* has not been reported as a proven vector of leishmaniasis; althought its role in disease transmission is debatable, the species is widely distributed in areas where the disease is endemic and has been found infected with *Endotrypanum* parasites[9]. Other sandfly species including *Lu. tortura* and *Lu. serrana* have also been found infected with parasites, but their role as vectors of the disease is not well understood.

We found that tuning Maxent parameters, specifically the regularization coefficient, provides better performance than using default parameters. This step appears to be important when calibrating models for species of public health concern considering that model fit appears to be sensitive to the regularization coefficient selected[69]. Additionally,

future climate models provide insights of the plausible climate conditions under different emission scenarios[50,52], however, such models may include climate conditions not present nowadays[61,62]. Hence, predicting vectors' suitability in novel climate conditions produces models with elevated uncertainty[61,62]. Here, we mitigated model predictions under novel environments via strict model transference and MOP analysis to identify areas with novel climates, where predictions should not be developed and conclusion should be considered with caution. Thus, we modified default parameters in Maxent via turning off extrapolation and clamping in the models, avoiding predictions in non-analogous novel climate conditions of future climate scenarios (Figs. S2), and restricting prediction to analogous climates only. This resulted in models with reduced uncertainty in future climate forecast and perilous prediction in novel climates lacking biological realism[24].

Finally, anticipating potential areas and populations at risk of exposure to disease vectors is a priority for effective disease control interventions[89]. While a previous study aimed to describe the occurrence of *Rhodnius ecuadoriensis* in two Ecuadorian provinces[90], to our knowledge, this is the first effort to model a complex ensemble of vector species under present-day and future climate conditions in Ecuador using remote sensing and future climate models (Fig. S3).

References

1. World Health Organization. *A global brief on vector-borne diseases*. (2014). Available at:

http://apps.who.int/iris/bitstream/10665/111008/1/WHO_DCO_WHD_2014.1_eng.pdf. (Accessed: 20th November 2015)

2. PAHO-CHA-CD. *Annual Country Report. Country or Territory Reporting VT in the Americas Between 2000-2015*. (Pan American Health Organization, 2015).

3. Mustafa, M. S., Rasotgi, V., Jain, S. & Gupta, V. Discovery of fifth serotype of dengue virus (DENV-5): A new public health dilemma in dengue control. *Med J Armed Forces India* **71,** 67–70 (2015).

4. San Martin, J. L. *et al.* The epidemiology of dengue in the Americas over the last three decades: A worrisome reality. *Am J Trop Med Hyg* **82,** 128–135 (2010).

5. Ministerio de Salud Pública. *Anuario Epidemiológico 1994-2014*. (2015). Available at: http://www.salud.gob.ec/direccion-nacional-de-vigilancia-epidemiologica/ (Accessed: 12th May 2016)

6. Bayot, B. & Barahona-Sanchez, M. G. Estudio de la Relacion entre la Variabilidad Oceano-atmosferica Local y Casos de Dengue en la Provincia de el Oro (Ecuador). (Escuela Superior Politecnica del Litoral, 2015).

7. Subsecretaria de Vigilancia de la Salud Pública. *Gaceta Epidemiológica Semanal No. 40*. (2015). Available at: http://instituciones.msp.gob.ec/images/Documentos/gaceta/Gaceta SE 40.pdf. (Accessed: 10th October 2015)

8. Subsecretaria de Vigilancia de la Salud Pública. Enfermedades transmitidas por vectores. Dengue. Ecuador, SE 1-35. *Dirección Nacional de Vigilancia Epidemiológica* (2016). Available at: http://www.salud.gob.ec/wp-

content/uploads/2013/02/DENGUE-GACETA-SE_35.pdf. (Accessed: 8th September 2016)

9. Gomez, E. A., Kato, H. & Hashiguchi, Y. Man-biting sand fly species and natural infection with the *Leishmania* promastigote in leishmaniasis-endemic areas of Ecuador. *Acta Trop* **140,** 41–49 (2014).

10. Kato, H. *et al.* First human cases of *Leishmania* (*Viannia*) *lainsoni* infection and a search for the vector sand flies in Ecuador. *PLoS Negl Trop Dis* **10,** e0004728 (2016).

11. Calvopina, M., Armijos, R. X. & Hashiguchi, Y. Epidemiology of leishmaniasis in Ecuador: Current status of knowledge - A review. *Mem Inst Oswaldo Cruz* **99,** 663–672 (2004).

12. Kato, H. *et al.* Detection and identification of Leishmania species within naturally infected sand flies in the andean areas of ecuador by a polymerase chain reaction. *Am J Trop Med Hyg* **72,** 87–93 (2005).

13. Hashiguchi, Y. *et al.* Andean leishmaniasis in Ecuador caused by infection with *Leishmania mexicana* and *L. major*-like parasites. *Am J Trop Med Hyg* **44,** 205–217 (1991).

14. Armijos, R. X. *et al.* Human cutaneous leishmaniasis in Ecuador: Identification of parasites by enzyme electrophoresis. *Am J Trop Med Hygene* **42,** 89–119 (1990).

15. Subsecretaria de Vigilancia de la Salud Pública. *Gaceta Epidemiológica Semanal No. 18*. (2016). Available at: http://www.salud.gob.ec/wp-content/uploads/2013/02/GACETA-GENERAL-SE18.pdf. (Accessed 8th

September 2016)

16. Le Ponti, F. *et al.* Leishmaniose en Equateur. 3. *Lutzomyia trapidoi*, vecteur de Leishmania panamensis. *Ann Soc Belge Med Trop* **74,** 23–28 (1994).

17. Le Ponti, F., Mouchet, J., Echeverria, R. & Guderian, R. H. Leishmaniose in Equateur. 2. Contacts homme/vecteurs de leishmaniose: Cas de *Lutzomyia trapidoi* et *Lu. gomezi*. *Ann Soc Belge Med Trop* **74,** 13–21 (1994).

18. Bright, E. A., Coleman, P. R., Rose, A. N. & Urban, M. L. LandScan 2011. *LandScan Ecuador* (2012).

19. Thiberville, S.-D. *et al.* Chikungunya fever: A clinical and virological investigation of outpatients on Reunion Island, South-West Indian Ocean. *PLoS Negl Trop Dis* **7,** e2004 (2013).

20. Escobar, L. E., Qiao, H. & Peterson, A. T. Forecasting Chikungunya spread in the Americas via data-driven, empirical approaches. *Parasit Vectors* **9,** 112 (2016).

21. Subsecretaria de Vigilancia de la Salud Pública. Enfermedades transmitidas por vectores. Zika Virus. Ecuador, SE 52-53, 2015. SE 1-35. (2016). *Dirección Nacional de Vigilancia Epidemiológica* (2016). Available at: http://www.salud.gob.ec/wp-content/uploads/2015/12/GACETA-ZIKA-SEM35.pdf. (Accessed: 8th September 2016)

22. Pan American Health Organization. *Technical Report: Recommendations for Scientific Evidence-Based Yellow Fever Risk Assessment in the Americas*. (PAHO, 2012).

23. Murray, C. J. L. & Lopez, A. D. Measuring the global burden of disease. *N Engl J*

Med **369,** 448–457 (2013).

24. Cifuentes, S. G. *et al.* Transition in the cause of fever from malaria to dengue, Northwestern Ecuador, 1990-2011. *Emerg Infect Dis* **19,** 1642–1645 (2013).

25. Carter, K. H. *et al.* Malaria in the Americas: Trends from 1959 to 2011. *Am J Trop Med Hyg* **92,** 302–316 (2015).

26. Aguilar, V. H. M., Abad-Franch, F., Racines, V. J. & Paucar, C. A. Epidemiology of chagas disease in Ecuador. A brief review. *Mem Inst Oswaldo Cruz* **94,** 387–393 (1999).

27. Guevara, Á. *et al.* First description of *Trypanosoma cruzi* human infection in Esmeraldas province, Ecuador. *Parasit Vectors* **7,** 358 (2014).

28. Grijalva, M. J. *et al.* Comprehensive survey of domiciliary triatomine species capable of transmitting chagas disease in southern Ecuador. *PLoS Negl Trop Dis* **9,** e0004142 (2015).

29. Guevara, A. G. *et al.* Seroepidemiological study of chagas disease in the southern Amazon region of Ecuador. *Trop Med Health* **41,** 21–25 (2013).

30. Cartelle Gestal, M., Holban, A. M., Escalante, S. & Cevallos, M. Epidemiology of tropical neglected diseases in Ecuador in the last 20 years. *PLoS ONE* **10,** e0138311 (2015).

31. Aguilar, H. M., Abad-Franch, F., Dias, J. C. P., Junqueira, A. C. V. & Coura, J. R. Chagas disease in the Amazon region. *Mem Inst Oswaldo Cruz* **102,** 47–56 (2007).

32. Quinde-Calderón, L., Rios-Quituizaca, P., Solorzano, L. & Dumonteil, E. Ten

years (2004-2014) of chagas disease surveillance and vector control in Ecuador: Successes and challenges. *Trop Med Int Heal* **21,** 84–92 (2016).

33. GBIF. Global Biodiversity Information Faclity. (2015). Available at: http://www.gbif.org/. (Accessed: 5th May 2015)

34. Centro de Referência em Informação Ambiental, C. speciesLink. (2015). Available at: http://splink.cria.org.br/. (Accessed: 5th May 2015)

35. Otegui, J., Ariño, A. H., Chavan, V. & Gaiji, S. On the dates of the GBIF mobilised primary biodiversity data records. *Biodiv Inf* **8,** 173–184 (2013).

36. Contreras Gutiérrez, M. A., Vivero, R. J., Vélez, I. D., Porter, C. H. & Uribe, S. DNA Barcoding for the identification of sand fly species (Diptera, Psychodidae, Phlebotominae) in Colombia. *PLoS ONE* **9,** e85496 (2014).

37. Pérez-Doria, A. & Bejarano, E. E. tRNASer (UCN) mitocondrial de *Lutzomyia hartmanni* prediccion de la estructura scundaria del tRNASer (UCN) mitocondrial del flebotomineo *Lutzomyia hartmanni* (Diptera:Psychodidae). *Acta Biol Colomb* **16,** 87–94 (2011).

38. Pinault, L. L. & Hunter, F. F. New highland distribution records of multiple *Anopheles* species in the Ecuadorian Andes. *Malar J* **10,** 236 (2011).

39. King, W. V. *Anopheles pseudopunctipennis*. *Science* **42,** 934–935 (1915).

40. Bargues, M. D. *et al.* Phylogeography and genetic variation of *Triatoma dimidiata*, the main chagas disease vector in Central America, and its position within the genus *Triatoma*. *PLoS Negl Trop Dis* **2,** e233 (2008).

41. Abad-Franch, F. *et al.* Biogeography of Triatominae (Hemiptera: Reduviidae) in

Ecuador: Implications for the design of control strategies. *Mem Inst Oswaldo Cruz* **96,** 611–620 (2001).

42. Kuwahara, K. *et al.* Genetic diversity of ribosomal RNA internal transcribed spacer sequences in *Lutzomyia* species from areas endemic for New World cutaneous leishmaniasis. *Acta Trop* **112,** 131–136 (2009).

43. Peterson, A. T. *et al. Ecological Niches and Geographic Distributions*. (Princeton University Press, 2011).

44. Barve, N. *et al.* The crucial role of the accessible area in ecological niche modeling and species distribution modeling. *Ecol Modell* **222,** 1810–1819 (2011).

45. Soberón, J. & Peterson, A. T. Interpretation of models of fundamental ecological niches and species' distributional areas. *Biodivers Informatics* **2,** 1–10 (2005).

46. Poo-Muñoz, D. A. *et al. Galictis cuja* (Mammalia): An update of current knowledge and geographic distribution. *Iheringia Série Zool* **104,** 341–346 (2014).

47. Mousson, L. *et al.* Phylogeography of *Aedes* (*Stegomyia*) *aegypti* (L.) and *Aedes* (*Stegomyia*) *albopictus* (Skuse) (Diptera: Culicidae) based on mitochondrial DNA variations. *Genet Res* **86,** 1–11 (2005).

48. Peterson, A. T. *Mapping Disease Transmission Risk: Enriching Models Using Biology and Ecology*. (Johns Hopkins University Press, 2014).

49. Hengl, T., Kilibarda, M., Carvalho-Ribeiro, E. D. & Reuter, H. I. Worldgrids — A public repository and a WPS for global environmental layers. *WorldGrids* (2015). Available at: http://worldgrids.org/doku.php?id=about&rev=1427534899. (Accessed: 1st January 2015)

50. Hijmans, R. J., Cameron, S. E., Parra, J. L., Jones, P. G. & Jarvis, A. Very high resolution interpolated climate surfaces for global land areas. *Int J Climatol* **25,** 1965–1978 (2005).

51. Qiao, H., Soberón, J., Escobar, L. E., Campbell, L. & Peterson, A. T. NicheA. Version 3.0.1. (2015). Available at: http://nichea.sourceforge.net/. (Accessed: 1st December 2015)

52. Kriticos, D. J. *et al.* CliMond: Global high-resolution historical and future scenario climate surfaces for bioclimatic modelling. *Methods Ecol Evol* **3,** 53–64 (2012).

53. Manning, M. R. *et al.* Misrepresentation of the IPCC CO2 emission scenarios. *Nat Geosci* **3,** 376–377 (2010).

54. Rahmstorf, S. *et al.* Recent climate observations compared to projections. *Science* **316,** 709 (2007).

55. Raupach, M. R. *et al.* Global and regional drivers of accelerating CO2 emissions. *Proc Natl Acad Sci USA* **104,** 10288–10293 (2007).

56. Riahi, K. *et al.* RCP 8.5-A scenario of comparatively high greenhouse gas emissions. *Clim Change* **109,** 33–57 (2011).

57. Snover, A. K., Mauger, G. S., Whitely, L. C., Krosby, M. & Tohver, I. in *Climate Change Impacts and Adaptation in Washington State: Technical Summaries for Decision Makers* (ed. Climate Impacts Group) (University of Washington, 2013).

58. Melillo, J., Richmond, T. C. & Yohe, G. in *Climate Change Impacts in the United States: The Third National Climate Assessment* 821–825 (U.S. Global Change Research Program, 2014).

59. Hutchinson, M., Xu, T., Houlder, D., Nix, H. & McMahon, J. *ANUCLIM 6.0 User's Guide*. (Australian National University, Fenner School of Environment and Society, 2009).

60. Kriticos, D. J., Jarošik, V. & Ota, N. Extending the suite of bioclim variables: A proposed registry system and case study using principal components analysis. *Methods Ecol Evol* **5,** 956–960 (2014).

61. Owens, H. L. *et al.* Constraints on interpretation of ecological niche models by limited environmental ranges on calibration areas. *Ecol Modell* **263,** 10–18 (2013).

62. Anderson, R. P. A framework for using niche models to estimate impacts of climate change on species distributions. *Ann N Y Acad Sci* **1297,** 8–28 (2013).

63. Elith, J., Kearney, M. & Phillips, S. J. The art of modelling range-shifting species. *Methods Ecol Evol* **1,** 330–342 (2010).

64. R Core Team. *R: A language and environment for statistical computing*. (R Foundation for Statistical Computing, 2016).

65. Phillips, S. J., Anderson, R. P. & Schapire, R. E. Maximum entropy modeling of species geographic distributions. *Ecol Modell* **190,** 231–259 (2006).

66. Elith, J. *et al.* A statistical explanation of Maxent for ecologists. *Divers Distrib* **17,** 43–57 (2011).

67. Merow, C., Smith, M. J. & Silander, J. A. A practical guide to MaxEnt for modeling species' distributions: what it does, and why inputs and settings matter. *Ecography* **36,** 1058–1069 (2013).

68. Phillips, S. J. & Dudík, M. Modeling of species distributions with Maxent: New

extensions and a comprehensive evaluation. *Ecography* **31,** 161–175 (2008).

69. Warren, D. L. & Seifert, S. N. Ecological niche modeling in Maxent: The importance of model complexity and the performance of model selection criteria. *Ecol Appl* **21,** 335–342 (2011).

70. Soberón, J. & Peterson, A. T. Ecological niche shifts and environmental space anisotropy: A cautionary note. *Rev Mex Biodivers* **82,** 1348–1355 (2011).

71. WorldPop. WorldPop Americas Alpha version 2010, 2015 and 2020 estimates of numbers of people per pixel. *Ecuador Population Map* (2015). Available at: http://www.worldpop.org.uk/data/summary/?contselect=America&countselect=Ecuador&typeselect=Population. (Accessed: 1st January 2016)

72. Colon-Gonzalez, F. J., Lake, I. R. & Bentham, G. Climate variability and dengue fever in warm and humid Mexico. *Am J Trop Med Hyg* **84,** 757–763 (2011).

73. Stewart-Ibarra, A. M. & Lowe, R. Climate and non-climate drivers of dengue epidemics in southern coastal Ecuador. *Am J Trop Med Hyg* **88,** 971–981 (2013).

74. Campbell, L. P. *et al.* Climate change influences on global distributions of dengue and chikungunya virus vectors. *Philos Trans R Soc B Biol Sci* **370,** 20140135 (2015).

75. Siraj, A. S. *et al.* Altitudinal changes in malaria incidence in highlands of Ethiopia and Colombia. *Science* **343,** 1154–1158 (2014).

76. Peterson, A. T. Shifting suitability for malaria vectors across Africa with warming climates. *BMC Infect Dis* **6,** 1–6 (2009).

77. Williams, J. W. & Jackson, S. T. Novel climates, no-analog communities, and

ecological surprises. *Front Ecol Environ* **5,** 475–482 (2007).

78. IPCC. *Impacts, Adaptation, and Vulnerability*. *Fifth Assessment Report of the Intergovernmental Panel on Climate Change* (Cambridge University Press, 2014).

79. Kadmon, R., Farber, O. & Danin, A. Effect of roadside bias on the accuracy of predictive maps produced by bioclimatic models. *Ecol Appl* **14,** 401–413 (2004).

80. Caminade, C. *et al.* Suitability of European climate for the Asian tiger mosquito *Aedes albopictus*: Recent trends and future scenarios. *J R Soc Interface* **9,** 2708–2717 (2012).

81. Fischer, D., Thomas, S. M., Neteler, M., Tjaden, N. B. & Beierkuhnlein, C. Climatic suitability of *Aedes albopictus* in Europe referring to climate change projections: Comparison of mechanistic and correlative niche modelling approaches. *Eurosurveillance* **19,** 1–13 (2014).

82. Medlock, J. M., Avenell, D., Barrass, I. & Leach, S. Analysis of the potential for survival and seasonal activity of *Aedes albopictus* (Diptera: Culicidae) in the United Kingdom. *J Vector Ecol* **31,** 292–304 (2006).

83. Peterson, A. T. Biogeography of diseases: A framework for analysis. *Naturwissenschaften* **95,** 483–491 (2008).

84. Estrada-Peña, A., Ostfeld, R. S., Peterson, A. T., Poulin, R. & de la Fuente, J. Effects of environmental change on zoonotic disease risk: An ecological primer. *Trends Parasitol* **30,** 205–214 (2014).

85. Escobar, L. E. *et al.* Ecology and geography of transmission of two bat-borne rabies lineages in Chile. *PLoS Negl Trop Dis* **7,** e2577 (2013).

86. Escobar, L. E., Juarez, C., Medina-Vogel, G. & Gonzales, C. M. First report on bat mortalities on wind farms in Chile. *Gayana* **79,** 11–17 (2015).

87. Zapata, S. *et al.* A study of a population of *Nyssomyia trapidoi* (Diptera: Psychodidae) caught on the Pacific coast of Ecuador. *Parasit Vectors* **5,** 144 (2012).

88. Takaoka, H., Gomez, E. A., Alexander, J. B. & Hashiguchi, Y. Natural infections with *Leishmania* promastigotes in *Lutzomyia ayacuchensis* (Diptera: Psychodidae) in an Andean focus of Ecuador. *J Med Entomol* **27,** 701–702 (1990).

89. Frieden, T. R. Government's role in protecting health and safety. *N Engl J Med* **368,** 1857–1859 (2013).

90. Yumiseva, C. A. Modelamiento Predictivo de Distribución de *Rhodnius ecuadoriensis*. Vector Principal de la Enfermedad de Chagas en Ecuador. (Thesis Magister, Universidad de San Francisco de Quito, 2014).

How to cite this article: Escobar, L. E. *et al.* Declining Prevalence of Disease Vectors Under Climate Change. *Sci. Rep.* **6**, 39150; doi: 10.1038/srep39150 (2016).
